# QTL Analysis of Adult Plant Resistance to Stripe Rust in a Winter Wheat Recombinant Inbred Population

**DOI:** 10.3390/plants10030572

**Published:** 2021-03-18

**Authors:** Kali M. Brandt, Xianming Chen, Javier F. Tabima, Deven R. See, Kelly J. Vining, Robert S. Zemetra

**Affiliations:** 1 Department of Biology, Clark University, Worcester, MA 01610, USA; JTabima@clarku.edu; 2 USDA-ARS Wheat Health, Genetics and Quality Research Unit and Plant Pathology, Washington State University, Pullman, WA 99164, USA; xianming.chen@usda.gov (X.C.); deven.see@usda.gov (D.R.S.); 3 Department of Horticulture, Oregon State University, Corvallis, OR 97331, USA; kelly.vining@oregonstate.edu; 4 Crop and Soil Science, Oregon State University, Corvallis, OR 97331, USA; robert.zemetra@oregonstate.edu

**Keywords:** wheat, quantitative trait loci (QTL), stripe rust, recombinant inbred line (RIL), genome wide association study (GWAS)

## Abstract

Stripe rust, caused by the fungus *Puccinia striiformis* f. sp. *tritici*, is a worldwide disease of wheat that causes devastating crop losses. Resistant cultivars have been developed over the last 40 years that have significantly reduced the economic impact of the disease on growers, but in heavy infection years it is mostly controlled through the intensive application of fungicides. The Pacific Northwest of the United States has an ideal climate for stripe rust and has one of the most diverse race compositions in the country. This has resulted in many waves of epidemics that have overcome most of the resistance genes traditionally used in elite germplasm. The best way to prevent high yield losses, reduce production costs to growers, and reduce the heavy application of fungicides is to pyramid multiple stripe rust resistance genes into new cultivars. Using genotyping-by-sequencing, we identified 4662 high quality variant positions in a recombinant inbred line population of 196 individuals derived from a cross between Skiles, a highly resistant winter wheat cultivar, and Goetze, a moderately to highly susceptible winter wheat cultivar, both developed at Oregon State University. A subsequent genome wide association study identified two quantitative trait loci (QTL) on chromosomes 3B and 3D within the predicted locations of stripe rust resistance genes. Resistance QTL, when combined together, conferred high levels of stripe rust resistance above the level of Skiles in some locations, indicating that these QTL would be important additions to future breeding efforts of Pacific Northwest winter wheat cultivars.

## 1. Introduction

**Figure 1:**
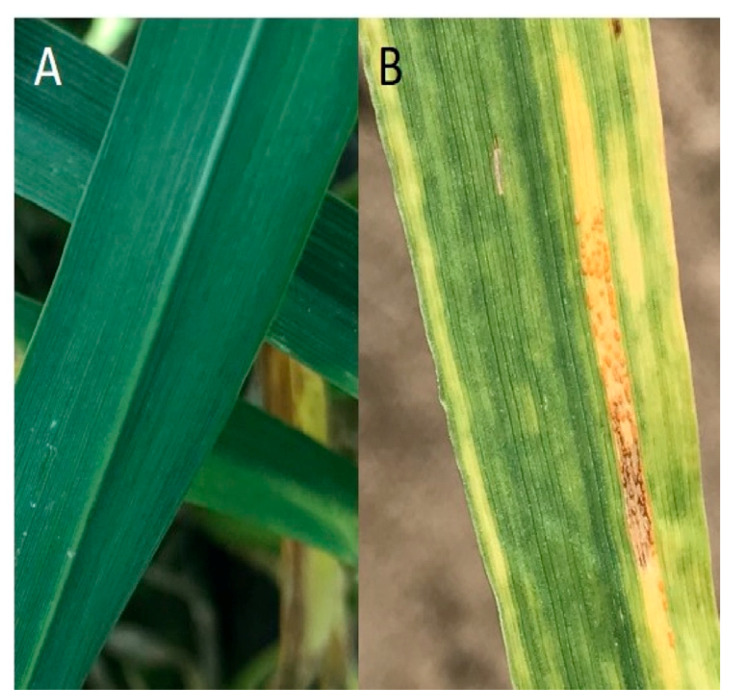
Wheat flag leaves with different levels of stripe rust infection. (**A**) A healthy, green flag leaf with no signs of infection. (**B**) A flag leaf infected with stripe rust. Chlorotic flecks and stripes indicate early infection symptoms. Orange uredinia in the chlorotic stripe are a classic sign of stripe rust. The fungus is also producing dark brown/black teliospores.

**Figure 2:**
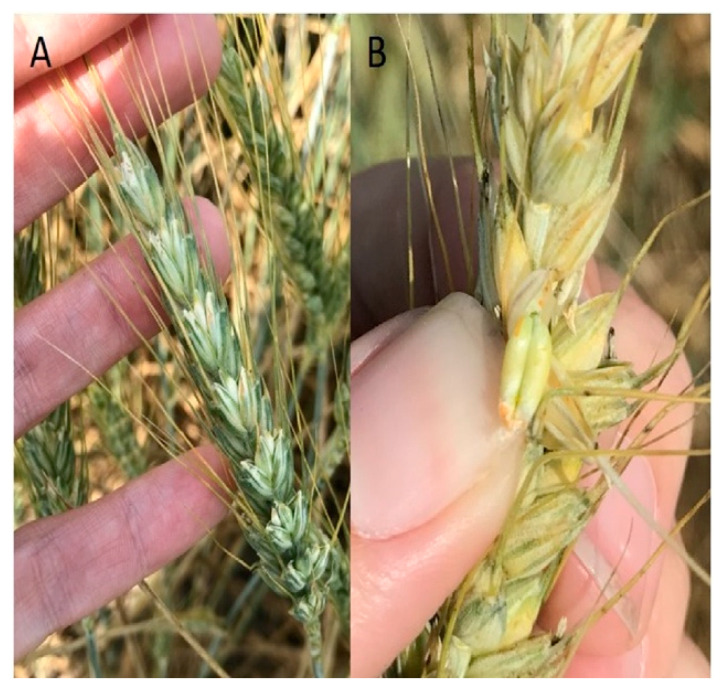
Stripe rust uredinia found on the heads of severely infected winter wheat plants. (**A**) Orange uredinia can be seen on the spikelets and on the awns of a severely infected plant. (**B**) This plant is so severely infected that uredinia have developed on the immature seed.

Stripe rust (*Puccinia striiformis* Westend. f. sp. *tritici* Erikss. (*Pst*)), also known as yellow rust, is a Basidiomycete fungal disease of wheat (*Triticum aestivum* L.) that causes significant yield losses around the world (Figure 1 and Figure 2) [1,2]. As of 2017, 88% of the world’s wheat production was susceptible to stripe rust, causing annual yield losses estimated to be worth USD 1 billion [3]. In the Pacific Northwest (PNW) of the United States, the climate is ideal for stripe rust, and this region has been battling yearly infections and frequent epidemics since the mid-twentieth century. Its mild wet winters and hot dry summers provide the ideal environment for *Pst* to infect, overwinter, oversummer, and reinfect [4,5]. Resistant cultivars deployed in the 1980s have largely been overcome by new races of *Pst* [4,6,7,8]. Therefore, foliar fungicides must be applied nearly every year on some varieties, and in heavy stripe rust years multiple applications of fungicide are used in an attempt to save the crop [4]. For example, in 2002, USD 2.5 million worth of fungicides were sprayed in the state of Washington alone to combat stripe rust infection [4]. Current resistance present in commercial cultivars has saved PNW growers hundreds of millions of dollars, but is still not sufficient during heavy stripe rust years [4]. The most effective way to reduce management costs, lower the negative environmental impacts, and improve food safety is to incorporate more durable sources of stripe rust resistance into commercial cultivars [1].

Stripe rust resistance comes in two forms, all stage resistance (ASR) and adult plant resistance (APR) [1]. ASR (also known as seedling resistance) genes are active during the entire life cycle of the plant, usually have a major effect, and are race specific; while APR genes are typically expressed when plants are older, APR genes are quantitative, usually have a minor effect, and are not race-specific [3,7,8,9]. ASR genes have an average life span of only 3.5 years when deployed as single genes in commercial cultivars, with some losing their resistance before commercial release [1,5,7,10,11,12,13]. APR genes are generally more durable than ASR genes, and while some can entirely inhibit sporulation of the stripe rust pathogen, they are less well characterized and can be difficult to select phenotypically [1,6,7,14].

In the PNW, new *Pst* races are identified each year, with a race composition that is more diverse than anywhere else in the country [5,15]. With the combination of favorable conditions for the pathogen and susceptible cultivars, complete yield loss has been documented in this region [6]. The use of resistant cultivars is able to reduce the potential yield loss to just over 8%, with chemical control reducing it even further [4,15]. However, chemical control still costs the growers millions of dollars each year, and a large number of the known stripe rust resistance genes have already become ineffective [5]. Due to this, breeders in the PNW have been focusing on pyramiding resistance genes by combining multiple resistance genes and types of resistance into individual cultivars to reduce the chances of the pathogen overcoming all genes [4,9,14,16]. Some studies have found that combining four or five APR genes with and without ASR genes can lead to near immunity [8,10,14]. One such cultivar that is highly resistant to stripe rust and grown in the PNW is “Skiles”, a soft white winter wheat released in 2008 that can be virtually free of infection in the field in some years [16,17].

Climate change, end-use quality demands, changing pathogen and pest pressures, and increasing population size all drive the demand for new cultivars [12,18]. This means that while Skiles has performed extremely well for stripe rust resistance in the PNW, growers and producers regularly require new cultivars. Therefore, it is important for breeders to be able to incorporate effective stripe rust resistance like that of Skiles into the next generation of commercial cultivars [1,4,6,13,19]. Traditionally, breeders used phenotypic selection in the field to determine disease resistance. Levels of APR resistance and stripe rust infection, however, are extremely sensitive to environmental factors such as moisture, temperature, and wind [5,9]. This sort of variation from year to year makes phenotypic selection difficult [20]. Therefore, a more efficient and effective way to incorporate these genes is by the use of marker assisted selection (MAS) [2,5,11,21].

MAS overcomes the need for consistent disease conditions and the testing of multiple races by allowing for direct selection on the genotype itself. Markers are DNA sequences that are linked to a trait of interest by a close genetic distance or are a part of the gene responsible for that trait [18]. The advent of whole genome sequencing via next generation sequencing (NGS) has provided a high-throughput process that generates thousands of single nucleotide polymorphisms (SNPs) which can be used as markers. SNPs that are found to be associated with stripe rust resistance can then be used to integrate these genes into new germplasm quickly and effectively [18,22]. One of the most efficient ways to identify SNPs associated with a trait of interest in a population is genotyping-by-sequencing (GBS) coupled with a genome-wide association study (GWAS) [12,23]. GBS is a reduced-representation genome genotyping technique widely used in plant species with highly complex genomes to obtain molecular markers using restriction enzymes [12]. GWAS is a quantitative analysis technique done to determine whether any SNPs are significantly associated with a certain trait, such as disease resistance [24,25]. Identification of SNPs using whole genome sequencing or GBS coupled with GWAS has been performed on many winter wheat cultivars in order to identify stripe rust resistance genes and quantitative trait loci (QTL), but many of these studies have not been replicated or validated, and new loci are being discovered regularly, meaning there are likely new sources of resistance that have yet to be characterized [5,13,18,23]. 

As mentioned previously, the commercial winter wheat cultivar Skiles is highly resistant to stripe rust, but all the genes responsible for this resistance need to be further characterized [16]. The cultivar “Goetze”, also a soft white winter wheat released by Oregon State University, was resistant to stripe rust at its release, but expected to have different resistance genes than Skiles [26]. It has become moderately to highly susceptible in the years since, though still expresses a low level of APR in some years. Identifying the sources of resistance in these lines will aid efforts to pyramid resistance genes, greatly increasing the durability of resistance in future PNW winter wheat cultivars and providing an economical benefit to the commercial growers that depend on this staple food crop. While there are hundreds of QTL associated with stripe rust resistance, they are widely dispersed across the world and most have not had reliable markers developed. Therefore, the goal of this study was to use GBS coupled with GWAS on a Skiles x Goetze RIL population to characterize the genes and/or QTL associated with their stripe rust resistances to be used for the development of markers for future breeding efforts in the PNW and beyond.

## 2. Results

### 2.1. Stripe Rust Phenotyping

**Figure 3:**
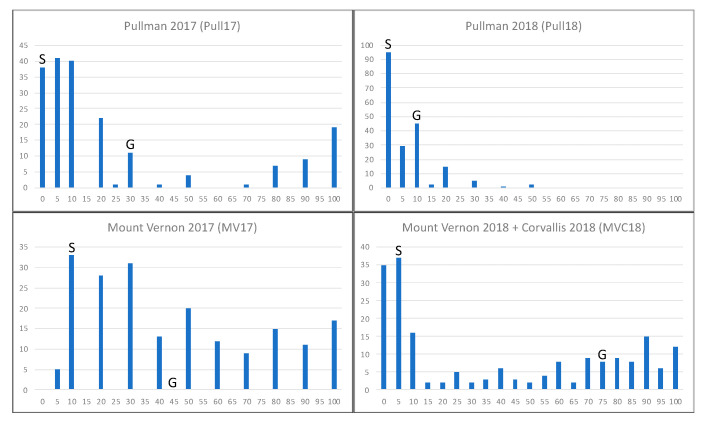
Histograms of the number of lines scored for each stripe rust severity in each treatment. Severity is on the horizontal axis and is a measure of the percentage of leaf area affected by the pathogen. The number of lines is on the vertical axis. “S” shows the severity score for Skiles in each treatment. “G” shows the severity score for Goetze in each treatment. The parental disease severity scores were not counted in the totals.

A total of 196 F_5_ recombinant inbred lines (RILs) from a cross between the cultivars Skiles and Goetze were evaluated for resistance to stripe rust in field nurseries in Pullman, WA, in 2017 and 2018; Mount Vernon, WA, in 2017 and 2018; and in Corvallis, OR, in 2018. Each location and year showed highly variable responses to stripe rust infection throughout the RIL population. Histograms of the number of lines scored at each value in each treatment are shown in Figure 3. Skiles received disease severity ratings of 0%, 0%, 10%, and 5% for Pullman 2017 (Pull17), Pullman 2018 (Pull18), Mount Vernon 2017 (MV17), and Mount Vernon 2018 + Corvallis 2018 (MVC18), respectively. Goetze received ratings of 30%, 10%, 45%, and 75% for Pull17, Pull18, MV17, and MVC18, respectively. In Pull17, 39 of the RILs were also rated at 0% infection like Skiles, and 155 lines had 30% or less infection, like Goetze. In Pull18, only 25 lines had higher severity values than Goetze. In MV17, five lines were scored at 5% severity, which is less than the resistant parent Skiles, and 33 lines scored the same 10% as Skiles. Overall in MV17, 111 lines scored lower than Goetze and 85 scored higher. In MVC18, 72 lines had severity equal to or lower than Skiles, and 58 lines had severity equal to or higher than Goetze. In Pull18, no lines had more than 50% severity. Conversely, in MV17, no lines were completely free of the disease.

### 2.2. SNP Genotyping

To identify the genetic components of resistance to stripe rust, all RILs were genotyped using genotyping-by-sequencing, with variants identified after mapping the resultant reads to the wheat reference genome sequence v1.0 (obtained from the International Wheat Genome Sequencing Consortium [27]). A total of 2,251,531 raw variants were identified by the Trait Analysis by aSSociation, Evolution and Linkage (TASSEL) GBS2 pipeline. Two lines were removed after filtering due to poor data quality and a high proportion of missing SNP calls. The unfiltered dataset had a GC content of 47.2%, with an average of 1.3 SNPs per 10Kb. The largest number of SNPs were mapped to chromosome 7B (173,679) and the smallest number to chromosome 6A (57,979). Postvariant call filtering per position resulted in 4662 high-quality filtered SNPs with a minor allele frequency (MAF) between 0.3 and 0.5, proportion missing between 0 and 0.086, and no heterozygous SNPs. The filtered dataset had a GC content of 49.9%, with an average of 0.003 SNPs per 10Kb. The largest number of SNPs were mapped to chromosome 3B (339) and the smallest number to chromosome 4D (78).

### 2.3. Statistical Analyses

To identify the statistical differences in stripe rust severity between locations and years, multiple models were fit to the data, with a linear mixed-effects model showing the best fit. The linear mixed-effects model results showed that in terms of stripe rust severity, the locations are significantly different (*p*-value < 1 × 10^−5^), the years are significantly different (*p*-value < 1 × 10^−5^), and the year by location interaction is significant (*p*-value <1 × 10^−5^) (Table S1). Due to the significant *p*-values of the years and locations, a Tukey’s HSD (honest significant difference) was performed to determine which treatments should be analyzed separately by the Genome Association and Prediction Integrated Tool (GAPIT). The results showed that each treatment is significantly different from the others (*p*-value <1 × 10^−7^, except for Corvallis 2018 and Mount Vernon 2018 (*p*-value = 0.99) (Table S2). Therefore, each significantly different treatment was analyzed separately in GAPIT, and Corvallis 2018 (Cor18) was combined with Mount Vernon 2018 (MV18) and analyzed as a single dataset referred to as MVC18.

**Figure 4:**
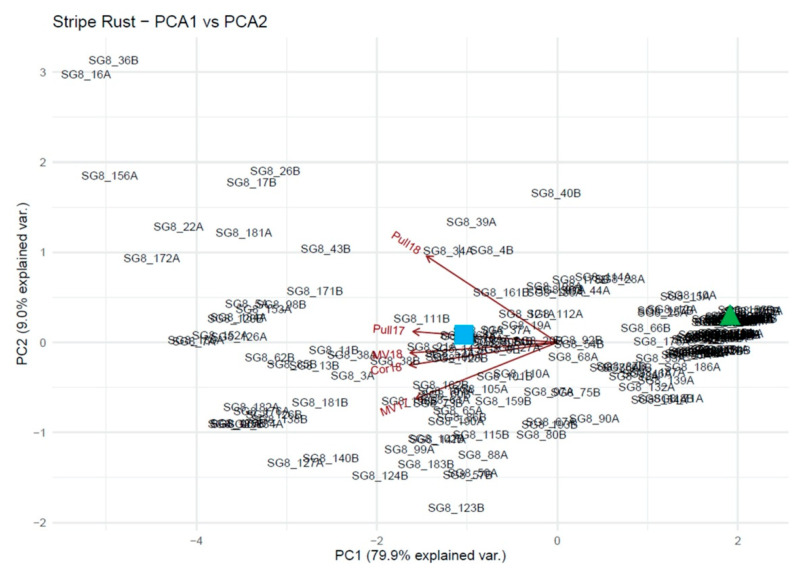
Principal component analysis representing the genetic diversity of the RIL population used in this study. Each principal component (PC) explains the corresponding contribution of each treatment to the PCs. PC1 explains 79.9% of the variation and PC2 explains 9.0% of the variation. The blue square represents Goetze, the moderately susceptible parent, located at approximately −1, 0 (PC1, PC2). The green triangle represents Skiles, the resistant parent, located at approximately 2, 0.25. The treatment vectors listed from top to bottom are Pullman 2018 (Pull18), Pullman 2017 (Pull17), Mount Vernon 2018 (MV18), Corvallis 2018 (Cor18), and Mount Vernon 2017 (MV17). In general, the individuals with high resistance in all treatments are located in the clusters to the right of the center. The most susceptible individuals are located in the top left corner, with various levels of susceptibility and resistance in between.

The PCA for the first two principal components is shown in Figure 4. Goetze is indicated by the blue square, and Skiles is indicated by the green triangle. The arrows show the contributions of the five treatments to the PCs. The tight cluster to the right that includes Skiles comprises lines that are highly resistant to stripe rust in all treatments. The rest of the lines are spread out based on varying resistance or susceptibility for each treatment, with the most overall susceptible lines being in the top left corner of the graph. PC1 explains 79.9% of the variation and PC2 explains 9.0% of the variation. Together they explain 88.9% of the variation and are sufficient for use in the GAPIT model for QTL discovery.

### 2.4. Genome Wide Association Study

**Figure 5:**
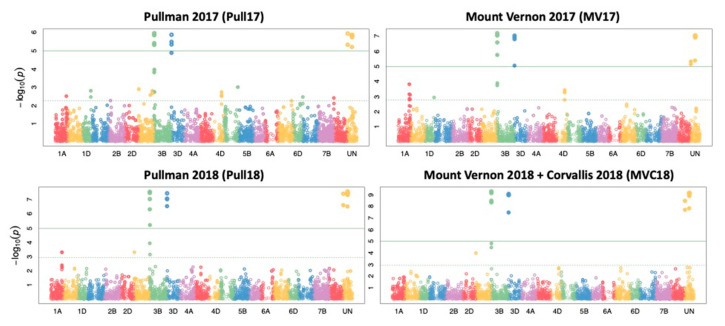
Manhattan plots for each treatment produced by GAPIT (Genome Association and Prediction Integrated Tool). The horizontal axis is the chromosome, with “UN” denoting a genomic scaffold that could not be mapped to any particular chromosome in the reference genome. The vertical axis is the *p*-value for each single nucleotide polymorphism (SNP)’s association with the trait of interest. The dotted horizontal line represents the –log10 (0.001) threshold. The solid green horizontal line represents the significant –log10 (*p*-value) threshold. All colored dots are SNPs predicted by TASSEL (Trait Analysis by aSSociation, Evolution and Linkage), and any dot above the solid green line is a significant SNP.

To identify SNPs with a genetic association to differences in stripe rust disease severity, a GWAS was performed for all RILs in each location and year. The Manhattan plots produced by the GWAS for each treatment are shown in Figure 5, with the solid green horizontal line indicating the threshold for significance. All points above the threshold line represent SNPs that are significantly associated with increased stripe rust resistance. Pullman 2017 returned 16 statistically significant SNPs: four on chromosome 3B, three on chromosome 3D, and nine on the Unidentified chromosome (denoting a genomic scaffold that could not be mapped to any particular chromosome in the reference genome). Pullman 2018 returned 18 significant SNPs: five on chromosome 3B, four on chromosome 3D, and nine on the Unidentified chromosome. Mount Vernon 2017 returned 17 significant SNPs: four on chromosome 3B, four on chromosome 3D, and nine on the Unidentified chromosome. Mount Vernon 2018 and Corvallis 2018 (MVC18) returned 17 significant SNPs: four on chromosome 3B, four on chromosome 3D, and nine on the Unidentified chromosome. A list of all unique significant SNPs identified by GAPIT, their locations on the reference genome assembly, *p*-value, R^2^ value, effect, and associated treatment are reported in Table 1. 

**Figure 6:**
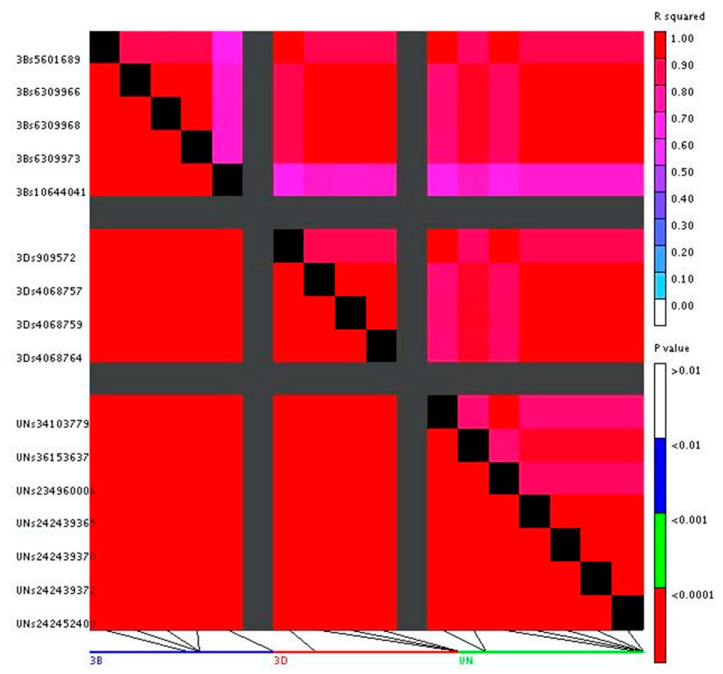
Heatmap of linkage disequilibrium (LD) for significant single nucleotide polymorphisms (SNPs). Tile colors represent the R^2^ value for each pairwise SNP (top right half), where R^2^ represents the degree of association between two SNPs. Significance of association between pairs of SNPs is represented in the bottom half of the figure (the *p*-value of the R^2^ value, legend in the bottom right). The horizontal line at the bottom is each chromosome with the identified SNPs being shown as black lines pointing to the corresponding placement on the plot.

The linkage disequilibrium (LD) values among all significant SNPs can be found in Supplemental Table S3. The subgroupings of SNPs as determined by these values are given here in Table 2. The graphical interpretation of the LD values among all significant SNPs is shown in Figure 6. An R^2^ of 1.0 indicates the two SNPs are completely linked, hence the state of one allele can predict the allelic state of the second position within this population. An R^2^ of 0.0 indicates that the two SNPs are completely unlinked from one another in this population. The LD values show that there are subgroups within the QTL that are highly linked with one another, but not with other QTL on the same chromosome. There are three subgroups of SNPs on chromosome 3B: Subgroup1 (S3B_6309966, S3B_6309968, and S3B_6309973), Subgroup2 (S3B_5601689 by itself), and Subgroup3 (S3B_10644041 by itself). There are two subgroups of SNPs on chromosome 3D: Subgroup1 (S3D_4068757, S3D_4068759, and S3D_4068764) and Subgroup2 (S3D_909572 by itself). There are three subgroups on the Unidentified chromosome: Subgroup1 (SUN_242439365, SUN_242439370, SUN_242439372, SUN_242452400, SUN_242452405, and SUN_242452407), Subgroup2 (SUN_34103779 and SUN_234960006), and Subgroup3 (SUN_36153637 by itself). Subgroup1 of 3B is highly correlated with Subgroup1 of 3D and Subgroup1 of the Unidentified chromosome (all with R^2^ of 1.0). Subgroup1 of 3D is also highly correlated with Subgroup1 of the Unidentified chromosome (R^2^ of 1.0). Subgroup2 of 3B is highly correlated with Subgroup2 of 3D and Subgroup2 of the Unidentified chromosome (all with R^2^ of 1.0). Subgroup3 of 3B is not highly correlated with any other subgroup or individual SNP. Subgroup3 of the Unidentified chromosome is highly correlated with Subgroup1 of 3B and 3D (R^2^ of between 0.95 and 0.96), and somewhat correlated with Subgroup2 of 3B and 3D (R^2^ of 0.89).

The top three lines that showed the highest level of resistance in every location and the bottom four lines that showed the lowest level of resistance in every location are shown in Table 3. The allelic state of each individual at each significant SNP is given, with “N” denoting an unknown allele. Unfortunately, the original Skiles and Goetze lines used to create this population were not retained and the plants used for genotyping were heterozygous at all significant loci. Therefore, they have not been included in Table 3.

### 2.5. Candidate Gene Analysis

**Figure 7:**
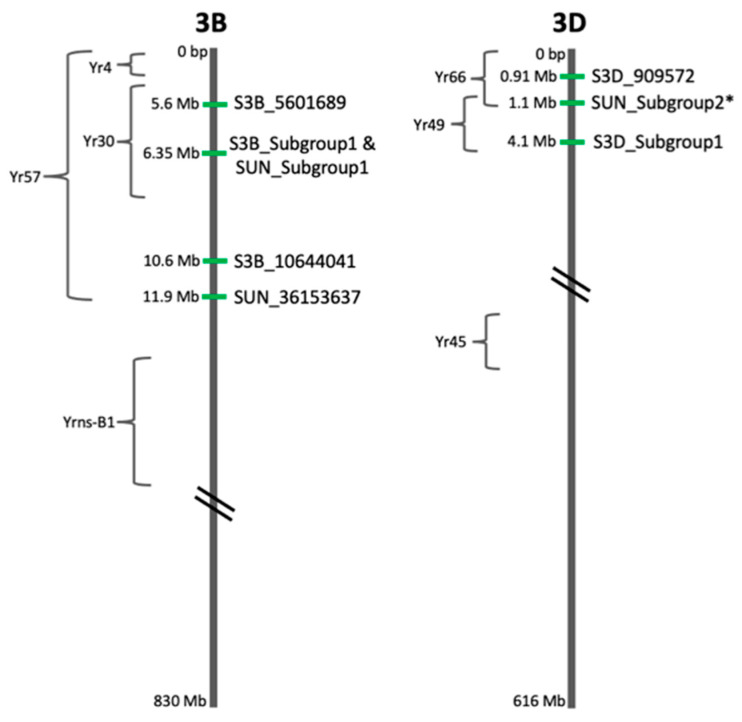
Location of the significant single nucleotide polymorphisms (SNPs) and their subgroups on the respective chromosomes as determined by the reference genome. The known stripe rust genes on each chromosome are also shown in their relative locations based on the GrainGenes maps. The double slash indicates the centromere. Chromosomes are not to scale. * SUN_Subgroup2 is not a highly confident placement, as the BLAST search of the related genes only matched this reference sequence location at 88%.

The placement of all significant SNPs and Subgroups of SNPs in relation to one another along with the putative location of named stripe rust genes is shown in Figure 7. The QTL identified on chromosome 3B are all located on the distal end of the short arm, between 5.6 and 12.3 Mb. SNP S3B_5601689 (Subgroup2) is located 216 bp downstream of gene TraesCS3B02G012400 at 5.6 Mb, which is predicted to code for a Knottin scorpion toxin-like superfamily that includes some plant defensins and antifungal proteins. SNPs S3B_6309966, S3B_6309968, and S3B_6309973 from chromosome 3B Subgroup1 are located approximately 260 bp from gene TraesCS3B02G015100 at 6.3 Mb, which is a pentatricopeptide repeat (PPR) gene in the PLN03218 superfamily. SNP S3B_10644041 (Subgroup3) is located in an untranslated region (UTR). The closest gene, TraesCS3B02G024600, is approximately 10,000 nucleotides away at 10.6 Mb. This gene is in the PLN02930 superfamily and is predicted to encode a serine exchange enzyme. Three stripe rust resistance genes, *Yr4*, *Yr30*, and *Yr57*, along with many QTL have also been mapped to this region of chromosome 3B, although the exact sequences and locations of these genes are unknown.

The QTL identified on chromosome 3D are all located on the distal end of the short arm, between 0.91 and 4.1 Mb. The SNPs in Subgroup1 of chromosome 3D (S3D_4068757, S3D_4068759, and S3D_4068764) are located in an intron of gene TraesCS3D02G011200, which is also a PPR repeat protein in the PLN03218 superfamily. The SNP in Subgroup2 of 3D, S3D_909572, is located in a UTR. The closest gene, TraesCS3D02G002000, is located approximately 16,000 nucleotides downstream at 0.93 Mb. It is in the Paf1 superfamily, which is an RNA polymerase II associated factor. The stripe rust resistance genes *Yr66* and *Yr49* have also been mapped to this region of chromosome 3D, although the exact sequences and locations are not known.

The QTL from the Unidentified chromosome segregate onto named chromosomes according to their subgroups. The SNPs from Subgroup2 (SUN_34103779 and SUN_234960006) are most likely located on chromosome 3D at approximately 1.1MB. SNP SUN_34103779 is located within gene TraesCSU02G041500, which is characterized as a leucine-rich repeat domain. The BLAST search of the gene’s protein sequence in Ensembl Plants returned a match of 88% for gene TraesCS3D02G002400 located at 1.1 Mb on chromosome 3D. SNP SUN_234960006 is located at gene TraesCSU02G162800, which is also a leucine-rich repeat domain. Performing a BLAST search in Ensembl Plants also returned an 88% match for gene TraesCS3D02G002400 on chromosome 3D. The other two subgroups from the Unidentified chromosome are most likely located on chromosome 3B. SNP SUN_36153637 (Subgroup3) is located in a UTR, with the closest gene, TraesCSU02G046100 located approximately 150,000 nucleotides away. This gene encodes a SINAT5 protein that functions in ubiquitin-mediated degradation resulting in the downregulation of auxin. Performing a BLAST of the protein sequence in Ensembl Plants returned a 99% match to gene TraesCS3B02G027400, located at 11.9 Mb on chromosome 3B. The SNPs in Subgroup1 (SUN_242439365, SUN_242439370, SUN_242439372, SUN_242452400, SUN_242452405, and SUN_242452407) are located close to genes TraesCSU02G166200 and TraesCSU02G166300, both of which are PPR repeat proteins in the PLN03218 superfamily. Performing a BLAST search for each gene returned the same result: a 100% match to gene TraesCS3B02G015100 located at 6.3 Mb on chromosome 3B. 

**Figure 8:**
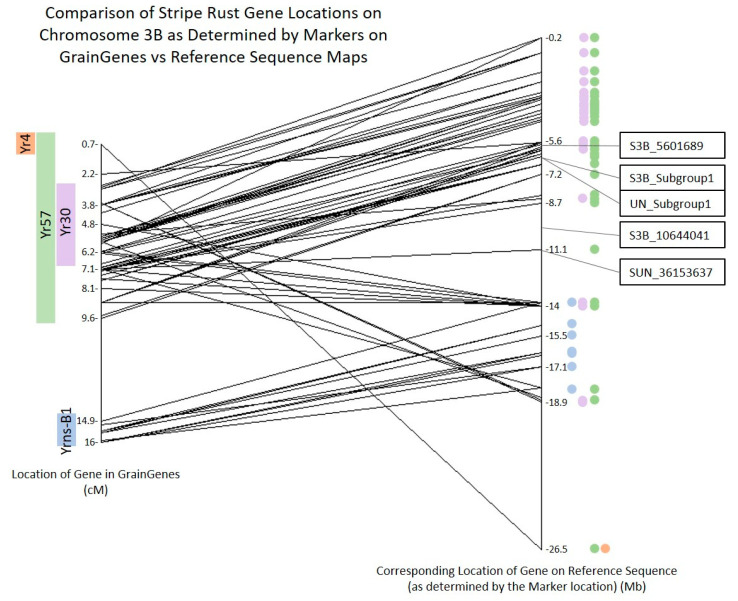
Chromosome 3B markers and their location as determined by the GrainGenes linkage map versus the reference sequence map RefSeq v1.0 (urgi.versailles.inra.fr). Markers from the GrainGenes “Wheat, *Yr* genes and QTL 3B” linkage map are arranged in map order with the linkage map location of the marker and putative stripe rust gene locations (cM) to the left. The corresponding location of that marker on the wheat reference genome (Mb) is on the right with a line connecting them. The colored circles represent the stripe rust gene that marker was associated with in the linkage map. The SNPs and/or Subgroups found in this study are to the right of the reference sequence location.

The USDA–ARS keeps a repository for wheat data called GrainGenes [28]. This site includes a linkage map of each wheat chromosome with known stripe rust genes and markers placed onto it. The wheat reference genome was published in August 2018, and the vast majority of data in GrainGenes precedes this [27]. Thus, the data in GrainGenes are based on linkage mapping and relative distances rather than precise physical locations. Therefore, all available marker sequences were mapped onto the reference genome in order to more accurately determine the placement of the SNPs and QTL found in this study. The placement of the markers and stripe rust genes from the GrainGenes linkage map for chromosome 3B is shown on the left side of Figure 8. The significant SNPs and subgroups identified in this study are shown on the right side of Figure 8. A list of all the marker names, stripe rust genes, GrainGenes locations, and reference sequence locations is available in Supplemental Table S4. SNP S3B_5601689 is located between markers IWB56857 and IWB8756 and within the potential location of *Yr57* and *Yr30*. The SNPs in Subgroup1 of both 3B and the Unidentified chromosome are located between markers IWB1837 and IWB23378, also within the potential location of *Yr57* and *Yr30*. SNP S3B_10644041 is located between markers IWA758 and IWB13827 within the potential location of *Yr30*. SNP SUN_36153637 was mapped between markers IWB57993 and IWB39411, also within the potential location of *Yr30*.

**Figure 9:**
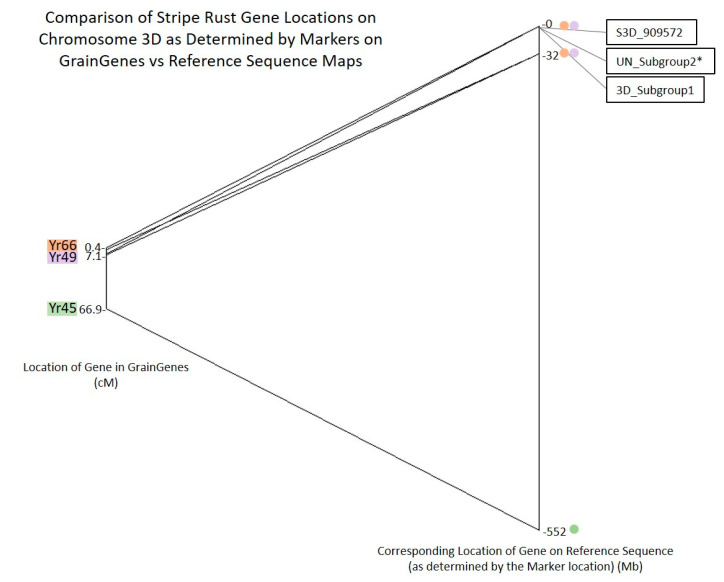
Chromosome 3D markers and their location as determined by the GrainGenes linkage map versus the reference sequence map RefSeq v1.0 (urgi.versailles.inra.fr). Markers from the GrainGenes “Wheat, *Yr* genes and QTL 3D” linkage map arranged in map order with the linkage map location of the marker and putative stripe rust gene locations (cM) to the left. The corresponding location of that marker on the wheat reference genome (Mb) is on the right with a line connecting them. The SNPs and/or Subgroups found in this study are to the right of the reference sequence location. * SUN_Subgroup2 is not a highly confident placement, as the BLAST search of the related genes only matched this reference sequence location at 88%.

The same process was repeated for the linkage map of chromosome 3D and is shown in Figure 9, with the list of marker names, stripe rust genes, GrainGenes locations, and reference sequence locations available in Supplemental Table S5. This chromosome is less well characterized than 3B and therefore contains significantly fewer markers. In GrainGenes, only *Yr45* is associated with any markers. *Yr49* and *Yr66* are somewhere on the distal end of the chromosome, upstream of the last marker IWA1123. Therefore, on the GrainGenes map in Figure 9, *Yr66* and *Yr49* are plotted using their putative linkage map locations rather than associated markers. On the reference sequence map on the right, their putative location is placed upstream of IWA1123, at 0 to 32 Mb. Upon reordering based on physical location, most markers remain in the same order, with the exceptions of IWA3573, IWA5030, and IWA6225. SNP S3D_909572 is located more than 32 Mb upstream of the first marker in GrainGenes and is within the potential region of both *Yr66* and *Yr49*. The SNPs in Subgroup1 of 3D are located at 4.1 Mb, approximately 29 Mb upstream of the closest marker in GrainGenes. The SNPs in Subgroup2 of the Unidentified chromosome are potentially located between these loci at 1.1 Mb, but the homology to this location was not as strong as the others in the Unidentified chromosome, so the location is not considered high confidence. *Yr45* is located across the centromere from these QTL.

## 3. Discussion

Stripe rust infection of winter wheat in the Pacific Northwest can cost growers millions of dollars every year and is primarily combated through fungicides and resistant cultivars. Fungicides are themselves costly, can be detrimental to the environment, and have the potential to lead to resistant strains of the pathogen. Resistant cultivars are the best option to fight the disease, but new sources of resistance are needed [1,4,5,13]. In this study we used a RIL population created with a highly resistant cultivar, Skiles, and a susceptible cultivar, Goetze. A GWAS using the genotype data from GBS and phenotypic data from multiple field locations identified two QTL with novel SNPs on chromosomes 3B and 3D. These QTL are within regions of the genome predicted to contain multiple stripe rust resistance genes [16].

Typically, biparental QTL mapping (BPQM), or linkage mapping, is performed on RIL populations such as the one used in this study [29,30]. However, when the original cross between Skiles and Goetze was made in 2009, the parents were not kept and propagated alongside the RILs. Therefore, no original parental genotype information was available in this population, and there is currently no established protocol for creating linkage maps without parental genotypes. For populations above 100 individuals, results from GWAS are comparable to results from BPQM when sufficient marker density is used [30,31]. GWAS also results in 10 to 200 times higher resolution than BPQM, which greatly facilitates gene discovery [29,30]. Famoso et al. 2011 utilized both techniques in rice and were able to reduce the candidate interval from 1720 kb containing 260 genes identified with BPQM to 81kb containing 13 genes identified with GWAS [30]. Multiple studies have also shown that GWAS and linkage mapping performed on a subset of the GWAS germplasm identify the same significant SNPs and QTL [32,33,34]. Therefore, due to the lack of parental data and the large number of individuals used, GWAS was considered an acceptable alternative for analyzing this population. 

Based on initial response to stripe rust in the field after its release, it was previously suspected that Skiles potentially carried the following resistance genes: *Yr4* (chromosome 3B), *Yr5* (2B), *Yr15* (1B), *Yr24* (1B), *Yr29* (1B), and/or *Yr32* (2A). In addition, based on field responses, it was thought that Goetze potentially carried the following resistance genes: *Yr4* (3B), *Yr5* (2B), *Yr10* (1B), *Yr16* (2D), *Yr17* (2A), *Yr29* (1B), and/or *Yr32* (2A). *Yr4* is an ASR gene. Although the disease severity data in this study were taken at the adult stage only, Skiles does not contain *Yr4* as it was previously shown to be susceptible to all five tested *Pst* races, including four races (PSTv-14, PSTv-37, PSTv-40, and PSTv-51) that are avirulent to Hybrid 46 carrying *Yr4,* as shown in other studies [16,35,36]. The identification of the 3B QTL in this study is consistent with the previous report of a QTL with major effect for high-temperature adult-plant (HTAP) resistance in Skiles mapped to 3B [16]. Therefore, it is highly unlikely that the QTL found on 3B is due to *Yr4* [15]. *Yr29* was thought to be the main contributor to Goetze’s resistance, but this gene is located on chromosome 1B, which was not identified as a source of resistance in this study.

In the field, RILs showed a wide range of resistance and susceptibility to the stripe rust pathogen (Figure 3). They also appear to exhibit transgressive segregation when compared to the parental lines, with some exhibiting extreme susceptibility and others almost complete resistance across locations and years. Pullman 2018 was an anomalous year in that no RIL showed a stripe rust severity score higher than 50%, and most lines were scored within the resistant ranges. In contrast, the susceptible check (PS279) in the nursery had 95 to 100% severity. In Pullman 2018, the most predominant race (PSTv-37) was present at approximately the same rate as in 2017 (48.7% and 57.1%, respectively), but there were an additional eight races found in 2018 compared to 2017 (X. Chen, personal communication). Therefore, it is possible that the additional *Pst* races in the 2018 Pullman field were not as virulent against Goetze’s resistance genes. At the time of its release, Goetze was considered resistant to stripe rust, but its resistance quickly waned (R. Zemetra, personal communication). Goetze is now considered a susceptible cultivar, although it sometimes behaves as a moderately susceptible cultivar with some HTAP resistance as seen in Mount Vernon and Pullman in 2017 where it received scores of 45% and 30%, respectively. The fact that Goetze only scored 10% in Pullman in 2018, RILs with higher severity scores were present, and the susceptible checks maintained 95 to 100% severity scores indicate that some of Goetze’s resistance genes are likely still effective against some races of the pathogen and can be useful for pyramiding. 

Five of the RILs showed the same high level of resistance as Skiles in every location and year. Seventeen RILs showed even higher resistance compared to Skiles in at least one treatment, including five lines that performed better in the most disease heavy treatment of Mount Vernon 2017 (Figure 3). Skiles is considered to be one of the most durable, highly resistant cultivars in the PNW. The fact that so many lines from this one cross with a moderately susceptible/susceptible cultivar consistently performed as well as or better than Skiles in a multitude of environments means that there is still room for improvement. This study also shows that the development of cultivars that are completely resistant to stripe rust without the need for any chemical treatments in average stripe rust years is realistic. Pyramiding more resistance genes on top of those that Skiles already contains will give growers an extremely reliable, resistant cultivar that will significantly reduce the amount of fungicides needed and improve yields and profits.

The publication of the wheat reference sequence in August of 2018 makes the ability to map SNPs and QTL more accurate than ever before [27]. However, the genetic map data generated before the release of the reference genome are based on linkage maps and relative distances rather than physical distances. In order to accurately map the QTL found in this study with relevant markers, the marker locations from GrainGenes were mapped to the reference genome where possible. Due to the nature of linkage mapping and the large LD decay of hexaploid wheat, many of the markers had been placed incorrectly in the GrainGenes maps, especially on chromosome 3B. It is clear that while the general trend of the physical locations matches the linkage map, there are many markers that are not in order, and some that are extremely distant from their original mapped location. One example of this disparity in locations is marker IWB63385. It is the first marker on the linkage map at 0.7 cM and is supposed to be the only marker within *Yr4*. Its location on the physical map, however, is much farther away and most likely around 10 Mb downstream of *Yrns-B1*. The new placement of this marker also stretches the potential location of *Yr57* across this whole section of the reference map, rather than just a subset. According to Liu et al. (2019), *Yr30* is tightly linked with Xgwm533, which is at 5.3 cM on the linkage map and 8.5 Mb in the reference sequence. Its physical location is further downstream on the reference sequence than most of its surrounding markers on the linkage map [16]. 

While the mapping of marker sequences to the reference is a relatively simple process, mapping the locations of the genes responsible for *Pst* resistance is not. The sequences of these resistance genes are unknown, and some of them are likely QTL rather than single genes, making their placement on the reference genome difficult. Therefore, their locations have been updated based on associated markers, but are still speculative rather than definite. Further characterization is needed to narrow down the actual locations and find highly reliable markers for each individual gene. This can be done with inoculations of RILs using *Pst* races with known interactions to specific stripe rust resistance genes. Targeted mutagenesis can also be used to knock out proteins within the suspected regions and then analyze the plants for stripe rust susceptibility changes. 

The QTL discovered in this study are located near to, or within, annotated genes. Five of the genes associated with these QTL also have functional annotations with disease resistance potential. Subgroup1 of chromosome 3D and Subgroup1 of chromosome 3B (which was determined to include Subgroup1 of the Unidentified chromosome) are characterized as pentatricopeptide repeat (PPR) genes. PPRs are tandem repeats of degenerate 35 amino acid motifs that are highly sequence specific. They are involved in many aspects of RNA metabolism and are extremely common in plant genomes, numbering in the hundreds [37]. This sequence specificity makes PPR genes good candidates to be used as reliable markers. The SNP S3B_5601689 is located just downstream of a gene in chromosome 3B that is predicted to be in the Knottin scorpion toxin-like superfamily, which includes plant defensin and antifungal proteins. Therefore, this gene may be a part of the *Yr57* or *Yr30* QTL and should be looked into in more detail. The SNPs from Subgroup2 of the Unidentified chromosome were tentatively mapped onto the distal end of chromosome 3D and are located within genes that possess six leucine-rich repeats (LRR) each. A leucine repeat is a conserved eleven-residue sequence motif of the form LxxLxLxxN/CxL that is usually involved in protein–protein interactions and sometimes associated with innate plant immunity to pathogens. These LRR domains are involved in detecting pathogen invasion either on the cell surface or intracellularly and then triggering downstream defenses. The majority of disease resistance genes encode leucine-rich repeat genes [38]. Therefore, these SNPs are likely to be directly associated with stripe rust resistance genes in this population and would be useful markers for breeding resistant cultivars. While these SNPs are potentially associated with either *Yr66* or *Yr49*, their exact location is not certain, and this should be resolved so that the resistance gene can be identified and characterized. These two SNPs were significant in every year and location, and therefore are likely important contributors to overall stripe rust resistance in this population.

A recent study by Liu et al. (2019) used Skiles-derived RILs and breeding lines to identify stripe rust resistance QTL on chromosomes 3B, 4B, 1B, 5A, 6B, and 7D [16]. Their ability to detect more QTL than this study is likely due to homozygosity between Skiles and Goetze at the resistance QTL locations of 4B, 1B, 5A, 6B, and 7D. Markers designed to the SNPs identified by Liu et al. (2019) for these locations showed the presence of the resistance alleles in both Skiles and Goetze, providing one explanation for why no QTL were identified in those regions in this study (data not published). Liu et al. (2019) did not identify a QTL on chromosome 3D, which suggests that this QTL could be coming from Goetze, as this cultivar was not an important part of their tested lines. The genotyping by Liu et al. (2019) was performed on a 90 K Illumina iSelect wheat SNP chip, which restricts the resulting loci to previously reported genomic locations [39]. Genotyping by sequencing done in this study allows for the discovery of novel SNPs, which could also explain the QTL on chromosome 3D. A study by Mu et al. (2020) identified a stripe rust resistance allele in Skiles on chromosome 3D using GWAS, but this allele was mapped to 26 Mb on the Chinese Spring reference genome and is therefore unlikely to be related to the QTL identified in this study [13]. Mu et al. (2020) also identified a resistance QTL on chromosome 3B, but it was mapped to 796 Mb on the long arm, while the 3B QTL from Liu et al. (2019) and the QTL from this study are on the short arm. The 3B QTL identified by Liu et al. (2019) was mapped to between 2.4 and 18.2 Mb, and the 3B QTL identified in this study was mapped to between 5.6 and 10.6 Mb. The SNPs in each study are not the same, likely due to the different genotyping methods used, but they may be identifying the same QTL.

Liu et al. (2019) concluded that their QTL on chromosome 3B was most likely a novel stripe rust resistance gene, as *Yr4* and *Yr57* are ASR genes, *Yrns-B1* is associated with marker Xgwm533, which is not linked to the resistance seen in their lines, and *Yr30* has been associated with a pseudo black chaff trait not seen in Skiles [40]. The QTL on 3B in this study are in the same general area as those in Liu et al. (2019), but the possibility of *Yr30* no longer being associated with pseudo black chaff should not be discounted, as all four QTL span a large portion of the potential location of *Yr30*. Therefore, the QTL found on chromosome 3B in this study is most likely associated with *Yr30* and/or a novel gene. 

The QTL found on chromosome 3D is located in a small region associated with *Yr49* and *Yr66*. *Yr66* has not been characterized and the earliest reference and all subsequently cited sources reference the Catalogue of Gene Symbols for Wheat: 2013–2014 Supplement, which only lists ”Bansal U 2014 Personal communication” as the source of the gene information [41,42,43]. As a result, it is not possible at this time to determine the likelihood of association between the QTL on chromosome 3D and this gene. *Yr49*, on the other hand, was characterized by Ellis et al. (2014) as a race-specific, adult plant resistance gene effective against every Australian *Pst* isolate [44]. As our QTL is associated with adult plant resistance and is located entirely within the predicted location of this gene, the QTL covering 0.91 to 4.1 Mb is likely *Yr49*. Since *Yr49* is known to be susceptible to Chinese races of *Pst* and resistant to Australian races, a greenhouse inoculation test can be performed to determine the validity of this hypothesis.

Unfortunately, in 2009 when the original cross between Skiles and Goetze was made, the parents were not kept and propagated alongside the RILs. The seed used in the field tests and in the DNA extraction for the parent lines was from foundation seed stocks. This resulted in both Skiles and Goetze being completely heterozygous at each SNP shown in Table 3. Therefore, it is not possible at this time to directly determine which QTL was contributed by which parent. However, there is a clear distinction between the allelic states at each locus and the associated stripe rust severity. The high levels of resistance which matched or exceeded Skiles appear to require the designated alleles at all SNP locations on both chromosomes 3B and 3D. The most susceptible lines from the least severe stripe rust treatment, Pullman 2018, also have the genotype of the most susceptible lines in Table 3; and the most resistant lines from the most severe treatment, Mount Vernon 2017, have the genotype of the most resistant lines. This result clearly indicates that these SNPs confer high levels of resistance to stripe rust in this germplasm. 

The resistance alleles on chromosome 3B are located in an area 13.9 Mb shorter than the GrainGenes markers associated with *Yr30*, and 10.8 Mb shorter than the SNPs identified by Liu et al. (2019). The resistance alleles on chromosome 3D are located in an area 28.8 Mb shorter than the GrainGenes markers associated with *Yr66* and *Yr49*. This represents a significant reduction in the length of genetic area that must be incorporated into the progeny of each cross in order to retain high levels of stripe rust resistance during the development of new varieties. Furthermore, treating each Subgroup and SNP with LD values of 1.0 between them as a single site for marker development, chromosome 3B would require only four markers to retain all 12 resistance alleles (S3B_5601689, S3B_Subgroup1+SUN_Subgroup1, S3B_10644041, and SUN_36153637), and chromosome 3D would require two markers to retain all six resistance alleles (S3D_909572+SUN_Subgroup2 and S3D_Subgroup1). Subsequent work will include developing Kompetitive Allele-Specific PCR (KASP, http://www.lgcgenomics.com) markers for each of the six locations on both chromosomes, and then using these for MAS development of cultivars with high levels of stripe rust resistance for use in the PNW (Table S6).

## 4. Materials and Methods

### 4.1. Germplasm

The population for this study consisted of 196 F_5_ recombinant inbred lines (RILs) from a cross between the cultivars Skiles (high levels of adult plant resistance) and Goetze (moderately to highly susceptible). Both Skiles and Goetze are soft white winter wheat varieties developed by Oregon State University and released in 2008 and 2007, respectively.

### 4.2. Experimental Design and Phenotyping

The 196 progeny lines plus lines of Skiles and Goetze were evaluated for stripe rust resistance in naturally infected field nurseries in Pullman, WA, in 2017 and 2018; Mount Vernon, WA, in 2017 and 2018; and in Corvallis, OR, in 2018. Mount Vernon and Pullman are approximately 500 km apart and have different weather conditions and races of *Pst* [15,16]. Each trial was planted in the fall of the year before the year indicated following common practices. A single replicate of each of the 196 RILs and two replicates of each parental line were planted in Mount Vernon and Pullman in 2017 and 2018 in a randomized design as single 50 cm rows with 20 cm between rows. PS270, an experimental winter wheat line without any resistance to stripe rust, was planted once every 20 rows and as border rows to increase stripe rust development in the Washington experiments. The nurseries in Washington were planted, maintained, and phenotyped by Xianming Chen’s team at the United States Department of Agriculture–Agricultural Research Service (USDA–ARS) and the Department of Plant Pathology at Washington State University. Two replicates of each RIL and four replicates of each parental line were planted in full 1.5 × 3 meter plots in Corvallis in 2018 in randomized complete blocks. Fertilization and weed control for all sites and years followed common practices. Stripe rust severity was assessed visually as the percentage of the leaf area infected at flowering (Zadoks GS60) in all locations in the spring of the year indicated [45]. 

### 4.3. DNA Extraction and Genotyping-By-Sequencing

Leaf tissue from parental lines and all 196 RILs was sampled in 2014 and the DNA extracted and purified by the Center for Genome Research and Biocomputing at Oregon State University. The DNA was then sent to the USDA–ARS Western Regional Small Grains Genotyping Laboratory at Washington State University in Pullman, WA, for library preparation utilizing *Pst*I and *Msp*I restriction enzymes for digestion and subsequent sequencing on the ion proton system (Thermo Fisher Scientific, Waltham, MA), with SNP variant calling performed as described by Merriman et al. (2012) [46] and Kohn et al. (2014) [47]. Raw reads with a length less than 64 bp were discarded. Variants in the population were discovered using the TASSEL (Trait Analysis by aSSociation Evolution and Linkage) 5.0 GBS2 pipeline [48]. A minimum of 10 total raw reads per tag was used as a threshold for quality control. The resultant tags were mapped against the wheat reference genome sequence v1.0 [27] using the Burrows–Wheeler aligner (BWA) and default parameters [49]. For the prediction of each variant, filtering was performed on the raw variants based on a minimum mapping quality (MQ) of five and a minimum base quality of 10. Postvariant prediction filtering was performed using the TASSEL 5.0 graphical interface to retain high-quality variants. Filtering was performed per variant site with a maximum threshold of 20% missing data per position, a minimum minor allele frequency (MAF) of 0.3, filtering out nonbiallelic SNPs, and indels marked as missing. Missing SNP calls were first imputed using the FILLIN option [50], using parental SNP calls and all options set to default parameters. Post imputation, all heterozygous SNP calls were marked as missing. The resulting dataset was used for all subsequent analyses.

### 4.4. Statistical Analyses

All statistical analyses were performed using R version 3.5 in RStudio version 1.1.463 [51]. This is the linear mixed-effects model used to determine the significance of the locations, years, and their interaction:

Disease Severity ~ Line + random (Location × Year)(1)

This was done using the function ”lme” with method ”REML” from the package ”nlme” [52]. A Tukey’s HSD (honest significant difference) test was then performed on the data in order to determine whether the disease severity scores between each treatment were significantly different. The Tukey’s HSD test was performed using the ”TukeyHSD” function in R. A principal component analysis (PCA) was also performed to determine the number of principal components necessary for the model to account for population structure. This was done using the ”prcomp” function and visualized in the ”ggbiplot” package [53].

### 4.5. Genome Wide Association Study and Candidate Gene Analysis

The GWAS was performed using the R package GAPIT V2. [54]. This package utilizes a compressed mixed linear model (CMLM) that accounts for population structure and kinship [55]. Population structure was accounted for by using the first two principal components in order to reduce the occurrence of spurious associations.

The linkage disequilibrium (LD) between significant SNPs was determined using TASSEL 5.0. The locations of the significant SNPs were then extracted from the GAPIT results. A region inclusive of two thousand base pairs on both sides of each SNP was searched for annotated genes using tools within Ensembl Plants [56]. The resulting gene set was further analyzed for predicted functions using the UniProt database [57] and the NCBI Conserved Domain Search [58]. 

GrainGenes, the USDA–ARS repository of wheat data, includes a linkage map of known stripe rust genes and markers. These maps predate the published wheat reference genome, however. Thus, the marker and gene placements in GrainGenes are based on relative distances rather than physical locations. In order to more accurately determine the placement of the QTL and significant SNPs found in this study with other markers and stripe rust genes, all available marker sequences from the ”Wheat, *Yr* genes and QTL 3B” linkage map between 0 and 12.2 cM, and 14.6 and 17.9 cM were used in a BLASTn (Basic Local Alignment Search Tool) against the RefSeq v1.0 chromosome 3B (urgi.versailles.inra.fr) to determine their physical location. Any result that was below a 98% match was not included. The resulting linkage map locations were then aligned by physical location on the reference sequence. The linkage map locations of stripe rust genes on chromosome 3B were also noted and subsequently changed to reflect the new marker placements on the reference genome. This procedure was then repeated for chromosome 3D with the ”Wheat, *Yr* genes and QTL 3D” map.

## 5. Conclusions

The use of GBS and the publication of the wheat reference genome have allowed for the discovery of novel QTL and SNPs in this population that are strongly associated with highly desirable stripe rust resistance packages that surpass even Skiles. The availability of the reference genome also allowed for the reordering of markers on chromosomes 3D and especially 3B that will help future efforts to pinpoint important stripe rust gene locations and narrow down the relevant marker distances in order to improve the efficacy of gene pyramiding. All QTL identified in this study were shown to be important for full resistance in each year and location, and the allelic state at each QTL segregated for resistance and susceptibility. These new markers combined with previously identified markers in the GrainGenes database can be used to further reduce the number and size of potential locations of important stripe rust resistance genes that have yet to be properly annotated so that they can be used in pyramiding efforts. The SNPs identified in this study can also be used immediately to incorporate elevated levels of resistance to stripe rust into elite germplasm, which would greatly benefit growers in this region. These varieties would require significantly less input of chemicals for controlling stripe rust, which would reduce costs, benefit the environment, and reduce the risk of creating fungicide-resistant *Pst* strains.

## Figures and Tables

**Table 1:** Single nucleotide polymorphisms (SNPs) significantly associated with stripe rust disease severity identified in the Manhattan plots. The “Chr” and “Position” columns correspond to the chromosome and position of the particular SNP in the reference sequence assembly in base pairs (bp). The “*p*-value” is the significance level from the Manhattan plots in Figure 5. “MAF” is the minor allele frequency at that SNP. The “R^2^ of Model without SNP” is the value of the coefficient of determination (R^2^) if the SNP were not present. The “R^2^ of Model with SNP” is the R^2^ value with that SNP included and indicates the effect of the quantitative trait loci (QTL). “Effect” is the effect of that allele on stripe rust disease severity.

Loc/Year	SNP	Chr	Position (bp)	*p*-Value	MAF	R^2^ of Model without SNP	R^2^ of Model with SNP	Effect
Pull17	S3B_5601689	3B	5601689	4.92E-06	0.4897	0.0293	0.1417	−14.4141
	S3B_6309966	3B	6309966	1.14E-06	0.4948	0.0293	0.1578	−15.5142
	S3B_6309968	3B	6309968	1.44E-06	0.4948	0.0293	0.1552	15.2734
	S3B_6309973	3B	6309973	3.97E-06	0.4923	0.0293	0.1441	−14.7001
	S3D_4068757	3D	4068757	4.67E-06	0.4923	0.0293	0.1423	−14.5157
	S3D_4068759	3D	4068759	1.37E-06	0.4948	0.0293	0.1558	15.3027
	S3D_4068764	3D	4068764	3.37E-06	0.5000	0.0293	0.1459	−14.7276
	SUN_234960006	UN	234960006	6.41E-06	0.4871	0.0293	0.1389	14.0261
	SUN_242439365	UN	242439365	1.40E-06	0.4974	0.0293	0.1556	15.3145
	SUN_242439370	UN	242439370	1.64E-06	0.4923	0.0293	0.1538	−15.2480
	SUN_242439372	UN	242439372	1.44E-06	0.4948	0.0293	0.1552	15.2734
	SUN_242452400	UN	242452400	1.44E-06	0.4948	0.0293	0.1552	15.2734
	SUN_242452405	UN	242452405	1.85E-06	0.4923	0.0293	0.1525	−15.0780
	SUN_242452407	UN	242452407	1.44E-06	0.4948	0.0293	0.1552	15.2734
	SUN_34103779	UN	34103779	4.82E-06	0.4871	0.0293	0.1420	−14.1982
	SUN_36153637	UN	36153637	1.18E-06	0.4923	0.0293	0.1575	15.2504
Pull18	S3B_10644041	3B	10644041	6.31E-06	0.4948	0.0454	0.1533	−3.6371
	S3B_5601689	3B	5601689	9.62E-08	0.4897	0.0454	0.1993	−4.5888
	S3B_6309966	3B	6309966	3.01E-08	0.4948	0.0454	0.2124	−4.8326
	S3B_6309968	3B	6309968	3.53E-08	0.4948	0.0454	0.2106	4.7814
	S3B_6309973	3B	6309973	5.02E-07	0.4923	0.0454	0.1808	−4.3634
	S3D_4068757	3D	4068757	9.81E-08	0.4923	0.0454	0.1990	−4.6159
	S3D_4068759	3D	4068759	3.80E-08	0.4948	0.0454	0.2098	4.7628
	S3D_4068764	3D	4068764	8.89E-08	0.5000	0.0454	0.2001	−4.6256
	S3D_909572	3D	909572	3.03E-07	0.4871	0.0454	0.1864	4.4075
	SUN_234960006	UN	234960006	3.19E-07	0.4871	0.0454	0.1858	4.3198
	SUN_242439365	UN	242439365	2.82E-08	0.4974	0.0454	0.2132	4.8227
	SUN_242439370	UN	242439370	4.26E-08	0.4923	0.0454	0.2085	−4.7693
	SUN_242439372	UN	242439372	3.53E-08	0.4948	0.0454	0.2106	4.7814
	SUN_242452400	UN	242452400	3.53E-08	0.4948	0.0454	0.2106	4.7814
	SUN_242452405	UN	242452405	4.94E-08	0.4923	0.0454	0.2068	−4.7150
	SUN_242452407	UN	242452407	3.53E-08	0.4948	0.0454	0.2106	4.7814
	SUN_34103779	UN	34103779	2.57E-07	0.4871	0.0454	0.1883	−4.3396
	SUN_36153637	UN	36153637	4.08E-08	0.4923	0.0454	0.2090	4.6933
MV17	S3B_5601689	3B	5601689	1.83E-06	0.4897	0.0399	0.1618	−13.6630
	S3B_6309966	3B	6309966	6.72E-08	0.4948	0.0399	0.1987	−15.7371
	S3B_6309968	3B	6309968	9.41E-08	0.4948	0.0399	0.1948	15.4645
	S3B_6309973	3B	6309973	2.71E-07	0.4923	0.0399	0.1829	−14.9769
	S3D_4068757	3D	4068757	1.64E-07	0.4923	0.0399	0.1886	−15.1775
	S3D_4068759	3D	4068759	9.68E-08	0.4948	0.0399	0.1945	15.4336
	S3D_4068764	3D	4068764	1.25E-07	0.5000	0.0399	0.1917	−15.3139
	S3D_909572	3D	909572	9.27E-06	0.4871	0.0399	0.1443	12.6911
	SUN_234960006	UN	234960006	4.29E-06	0.4871	0.0399	0.1526	12.9439
	SUN_242439365	UN	242439365	1.24E-07	0.4974	0.0399	0.1918	15.3257
	SUN_242439370	UN	242439370	1.26E-07	0.4923	0.0399	0.1915	−15.3581
	SUN_242439372	UN	242439372	9.41E-08	0.4948	0.0399	0.1948	15.4645
	SUN_242452400	UN	242452400	9.41E-08	0.4948	0.0399	0.1948	15.4645
	SUN_242452405	UN	242452405	1.11E-07	0.4923	0.0399	0.1929	−15.3378
	SUN_242452407	UN	242452407	9.41E-08	0.4948	0.0399	0.1948	15.4645
	SUN_34103779	UN	34103779	7.49E-06	0.4871	0.0399	0.1466	−12.5548
	SUN_36153637	UN	36153637	4.92E-06	0.4923	0.0399	0.1511	12.9378
MVC18	S3B_5601689	3B	5601689	4.87E-09	0.4897	0.1426	0.3115	−22.4636
	S3B_6309966	3B	6309966	5.65E-10	0.4948	0.1426	0.3344	−24.5208
	S3B_6309968	3B	6309968	7.64E-10	0.4948	0.1426	0.3312	24.2253
	S3B_6309973	3B	6309973	3.51E-09	0.4923	0.1426	0.3150	−23.3131
	S3D_4068757	3D	4068757	1.14E-09	0.4923	0.1426	0.3269	−23.7960
	S3D_4068759	3D	4068759	1.17E-09	0.4948	0.1426	0.3266	23.7896
	S3D_4068764	3D	4068764	9.19E-10	0.5000	0.1426	0.3292	−23.8661
	S3D_909572	3D	909572	3.61E-08	0.4871	0.1426	0.2908	20.9861
	SUN_234960006	UN	234960006	1.57E-08	0.4871	0.1426	0.2993	21.2737
	SUN_242439365	UN	242439365	9.10E-10	0.4974	0.1426	0.3293	24.0836
	SUN_242439370	UN	242439370	1.14E-09	0.4923	0.1426	0.3269	−24.0405
	SUN_242439372	UN	242439372	7.64E-10	0.4948	0.1426	0.3312	24.2253
	SUN_242452400	UN	242452400	7.64E-10	0.4948	0.1426	0.3312	24.2253
	SUN_242452405	UN	242452405	1.35E-09	0.4923	0.1426	0.3251	−23.7279
	SUN_242452407	UN	242452407	7.64E-10	0.4948	0.1426	0.3312	24.2253
	SUN_34103779	UN	34103779	2.13E-08	0.4871	0.1426	0.2962	−20.8097
	SUN_36153637	UN	36153637	3.62E-09	0.4923	0.1426	0.3146	22.5449

**Table 2:** The groupings of significant single nucleotide polymorphisms (SNPs) on each chromosome as determined by their linkage disequilibrium values. UN refers to the Unidentified chromosome. An R^2^ of 1.0 indicates that the SNPs are completely correlated with one another in this population, meaning the presence of one allele can predict the allelic state of the other(s).

Chromosome	Subgroup	Significant SNP	R^2^ Within Subgroup	R^2^ of 1.0 With Other Subgroups
3B	Subgroup1	S3B_6309966	1.0	3D Subgroup1 UN Subgroup1
		S3B_6309968		
		S3B_6309973		
	Subgroup2	S3B_5601689		3D Subgroup2 UN Subgroup2
	Subgroup3	S3B_10644041		
3D	Subgroup1	S3D_4068757	1.0	3B Subgroup1 UN Subgroup1
		S3D_4068759		
		S3D_4068764		
	Subgroup2	S3D_909572		3B Subgroup2 UN Subgroup2
UN	Subgroup1	SUN_242439365	1.0	3B Subgroup1 3D Subgroup1
		SUN_242439370		
		SUN_242439372		
		SUN_242452400		
		SUN_242452405		
		SUN_242452407		
	Subgroup2	SUN_34103779	1.0	3B Subgroup2 3D Subgroup2
		SUN_234960006		
	Subgroup3	SUN_36153637		

**Table 3:** Significant SNPs from all locations and years (Loc/Year) and their allelic state in the most resistant lines and the most susceptible lines. The stripe rust severity score of each line for each Loc/Year is given in the columns to the right. “N” means the allele at that location is unknown. The significant SNPs are grouped according to their respective subgroups.

	Line	SG8_163A	SG8_179B	SG8_195A	SG8_156A	SG8_172A	SG8_180A	SG8_7A
**Significant SNPs**	3B_5601689	C	C	N	A	A	A	A
	3B_6309966	G	G	G	C	C	C	C
	3B_6309968	A	A	A	G	G	G	G
	3B_6309973	G	G	G	C	C	C	C
	UN_242439365	C	C	C	G	G	G	G
	UN_242439370	C	C	C	G	G	G	G
	UN_242439372	T	T	T	C	C	C	C
	UN_242452400	C	C	C	G	G	G	G
	UN_242452405	T	T	T	C	C	C	C
	UN_242452407	C	C	C	G	G	G	G
	3B_10644041	T	T	T	A	A	A	A
	UN_36153637	C	C	C	T	T	T	T
	3D_909572	G	G	N	T	T	T	T
	UN_34103779	G	G	N	A	A	A	A
	UN_234960006	C	C	N	T	T	T	T
	3D_4068757	G	G	G	C	C	C	C
	3D_4068759	A	A	A	G	G	G	G
	3D_4068764	G	G	G	C	C	C	C
**Disease Severity Scores**	Pull17	0	0	0	100	100	100	100
	Pull18	0	0	0	40	30	20	20
	MV17	5	5	5	100	100	100	100
	MVC18	0	0	0	100	100	100	100
		Top 3 resistant in every location			Bottom 4 susceptible in every location			

## Data Availability

Raw sequencing data of the RILs have been deposited in the NCBI SRA (https://www.ncbi.nlm.nih.gov/sra) under the BioProject accession number PRJNA610805. Phenotypes and marker information are available on the Open Science Framework (OSF) (https://osf.io), under DOI 10.17605/OSF.IO/JWF3B.

## Supplementary Materials

The following are available online at https://www.mdpi.com/2223-7747/10/3/572/s1, Table S1: Linear mixed-effects model of disease severity, Table S2: Tukey’s HSD (honestly significant difference) test of all treatments, Table S3: Linkage disequilibrium (LD) analysis of all significant SNPs and their corresponding R^2^ values, Table S4: Stripe rust markers from GrainGenes chromosome 3B with their linkage map location, reference genome location, and associated stripe rust genes, Table S5: Stripe rust markers from GrainGenes chromosome 3D with their linkage map location, reference genome location, and associated stripe rust genes, Table S6: Unvalidated KASP marker designs.
