# Supplementary material for: QTL Analysis of Adult Plant Resistance to Stripe Rust in a Winter Wheat Recombinant Inbred Population
Source: Plants (Basel). 2021 Mar 18;10(3):572. doi: 10.3390/plants10030572 (PMC8002966; doi:10.3390/plants10030572)
Supplement: Supplementary file 1 [file plants-10-00572-s001.zip › Table S1.docx]

Table S1: Linear mixed-effects model of disease severity as a function of Year, Location, and Year by Location interaction (Year*Location). The *p*-values indicate that all have significant effect on the Disease Severity.

| **Linear mixed-effects model fit by REML** | | | | | |
| --- | --- | --- | --- | --- | --- |
|  | Value | Standard Error | Degrees of Freedom | *t*-value | *p*-value |
| (Intercept) | 713.0434 | 75.60742 | 965 | 9.430865 | <1e-5*** |
| Year | -38.2354 | 4.26776 | 965 | -8.959122 | <1e-5*** |
| Location | -384.2156 | 43.96319 | 965 | -8.739484 | <1e-5*** |
| Year*Location | 21.5018 | 2.46303 | 965 | 8.729788 | <1e-5*** |
