# Supplementary material for: QTL Analysis of Adult Plant Resistance to Stripe Rust in a Winter Wheat Recombinant Inbred Population
Source: Plants (Basel). 2021 Mar 18;10(3):572. doi: 10.3390/plants10030572 (PMC8002966; doi:10.3390/plants10030572)
Supplement: Supplementary file 1 [file plants-10-00572-s001.zip › Table S2.docx]

Table S2: Tukey’s HSD (honestly significant difference) Test of all Treatments compared to one another. The ‘*p* adjusted’ value shows that all treatments are significantly different from one another, expect for Corvallis 2018 (Cor18) and Mount Vernon 2018 (MV18). Therefore, disease severity scores for Corvallis 2018 will be combined with scores from Mount Vernon 2018 for GWAS analysis. The others will be kept separate.

| **Tukey’s HSD** | | | | |
| --- | --- | --- | --- | --- |
| Treatment | Diff | Lower | Upper | *p* adjusted |
| MV18 – MV17 | -4.6616162 | -6.773765 | -2.549468 | 0.0000001*** |
| Pull17 – MV17 | -18.8383838 | -20.950532 | -16.726235 | 0.0000000*** |
| Pull18 – MV17 | -38.0050505 | -40.117199 | -35.892902 | 0.0000000*** |
| Cor18 – MV17 | -4.9873737 | -6.816548 | -3.158200 | 0.0000000*** |
| Pull17 – MV18 | -14.1767677 | -16.288916 | -12.064619 | 0.0000000*** |
| Pull18 – MV18 | -33.3434343 | -35.455583 | -31.231286 | 0.0000000*** |
| Cor18 – MV18 | -0.3257576 | -2.154932 | 1.503417 | 0.9881925 |
| Pull18 – Pull17 | -19.1666667 | -21.278815 | -17.054518 | 0.0000000*** |
| Cor18 – Pull17 | 13.8510101 | 12.021836 | 15.680184 | 0.0000000*** |
| Cor18 – Pull18 | 33.0176768 | 31.188503 | 34.846851 | 0.0000000*** |
