# Supplementary material for: QTL Analysis of Adult Plant Resistance to Stripe Rust in a Winter Wheat Recombinant Inbred Population
Source: Plants (Basel). 2021 Mar 18;10(3):572. doi: 10.3390/plants10030572 (PMC8002966; doi:10.3390/plants10030572)
Supplement: Supplementary file 1 [file plants-10-00572-s001.zip › Table S3.docx]

Table S3: Linkage disequilibrium (LD) analysis of all significant SNPs and their corresponding R^2^ values. An R^2^ of 1.0 indicates the two SNPs are completely correlated with one another in this population. An R^2^ of 0.0 indicates that the two SNPs are completely unlinked from one another in this population.

| **Locus1** | **Position1** | **States1** | **Locus2** | **Position2** | **States2** | **R^2^** |
| --- | --- | --- | --- | --- | --- | --- |
| 3B | 6309968 | G:A | 3B | 6309966 | C:G | 1.00 |
| 3B | 6309973 | C:G | 3B | 6309966 | C:G | 1.00 |
| 3B | 6309973 | C:G | 3B | 6309968 | G:A | 1.00 |
| 3D | 909572 | G:T | 3B | 5601689 | C:A | 1.00 |
| 3D | 4068757 | C:G | 3B | 6309966 | C:G | 1.00 |
| 3D | 4068757 | C:G | 3B | 6309968 | G:A | 1.00 |
| 3D | 4068757 | C:G | 3B | 6309973 | C:G | 1.00 |
| 3D | 4068759 | G:A | 3B | 6309966 | C:G | 1.00 |
| 3D | 4068759 | G:A | 3B | 6309968 | G:A | 1.00 |
| 3D | 4068759 | G:A | 3B | 6309973 | C:G | 1.00 |
| 3D | 4068759 | G:A | 3D | 4068757 | C:G | 1.00 |
| 3D | 4068764 | C:G | 3B | 6309966 | C:G | 1.00 |
| 3D | 4068764 | C:G | 3B | 6309968 | G:A | 1.00 |
| 3D | 4068764 | C:G | 3B | 6309973 | C:G | 1.00 |
| 3D | 4068764 | C:G | 3D | 4068757 | C:G | 1.00 |
| 3D | 4068764 | C:G | 3D | 4068759 | G:A | 1.00 |
| UN | 34103779 | G:A | 3B | 5601689 | C:A | 1.00 |
| UN | 34103779 | G:A | 3D | 909572 | G:T | 1.00 |
| UN | 234960006 | C:T | 3B | 5601689 | C:A | 1.00 |
| UN | 234960006 | C:T | 3D | 909572 | G:T | 1.00 |
| UN | 234960006 | C:T | UN | 34103779 | G:A | 1.00 |
| UN | 242439365 | G:C | 3B | 6309966 | C:G | 1.00 |
| UN | 242439365 | G:C | 3B | 6309968 | G:A | 1.00 |
| UN | 242439365 | G:C | 3B | 6309973 | C:G | 1.00 |
| UN | 242439365 | G:C | 3D | 4068757 | C:G | 1.00 |
| UN | 242439365 | G:C | 3D | 4068759 | G:A | 1.00 |
| UN | 242439365 | G:C | 3D | 4068764 | C:G | 1.00 |
| UN | 242439370 | C:T | 3B | 6309966 | C:G | 1.00 |
| UN | 242439370 | C:T | 3B | 6309968 | G:A | 1.00 |
| UN | 242439370 | C:T | 3B | 6309973 | C:G | 1.00 |
| UN | 242439370 | C:T | 3D | 4068757 | C:G | 1.00 |
| UN | 242439370 | C:T | 3D | 4068759 | G:A | 1.00 |
| UN | 242439370 | C:T | 3D | 4068764 | C:G | 1.00 |
| UN | 242439370 | C:T | UN | 242439365 | G:C | 1.00 |
| UN | 242439372 | G:C | 3B | 6309966 | C:G | 1.00 |
| UN | 242439372 | G:C | 3B | 6309968 | G:A | 1.00 |
| UN | 242439372 | G:C | 3B | 6309973 | C:G | 1.00 |
| UN | 242439372 | G:C | 3D | 4068757 | C:G | 1.00 |
| UN | 242439372 | G:C | 3D | 4068759 | G:A | 1.00 |
| UN | 242439372 | G:C | 3D | 4068764 | C:G | 1.00 |
| UN | 242439372 | G:C | UN | 242439365 | G:C | 1.00 |
| UN | 242439372 | G:C | UN | 242439370 | C:T | 1.00 |
| UN | 242452400 | G:C | 3B | 6309966 | C:G | 1.00 |
| UN | 242452400 | G:C | 3B | 6309968 | G:A | 1.00 |
| UN | 242452400 | G:C | 3B | 6309973 | C:G | 1.00 |
| UN | 242452400 | G:C | 3D | 4068757 | C:G | 1.00 |
| UN | 242452400 | G:C | 3D | 4068759 | G:A | 1.00 |
| UN | 242452400 | G:C | 3D | 4068764 | C:G | 1.00 |
| UN | 242452400 | G:C | UN | 242439365 | G:C | 1.00 |
| UN | 242452400 | G:C | UN | 242439370 | C:T | 1.00 |
| UN | 242452400 | G:C | UN | 242439372 | G:C | 1.00 |
| UN | 242452405 | C:T | 3B | 6309966 | C:G | 1.00 |
| UN | 242452405 | C:T | 3B | 6309968 | G:A | 1.00 |
| UN | 242452405 | C:T | 3B | 6309973 | C:G | 1.00 |
| UN | 242452405 | C:T | 3D | 4068757 | C:G | 1.00 |
| UN | 242452405 | C:T | 3D | 4068759 | G:A | 1.00 |
| UN | 242452405 | C:T | 3D | 4068764 | C:G | 1.00 |
| UN | 242452405 | C:T | UN | 242439365 | G:C | 1.00 |
| UN | 242452405 | C:T | UN | 242439370 | C:T | 1.00 |
| UN | 242452405 | C:T | UN | 242439372 | G:C | 1.00 |
| UN | 242452405 | C:T | UN | 242452400 | G:C | 1.00 |
| UN | 242452407 | G:C | 3B | 6309966 | C:G | 1.00 |
| UN | 242452407 | G:C | 3B | 6309968 | G:A | 1.00 |
| UN | 242452407 | G:C | 3B | 6309973 | C:G | 1.00 |
| UN | 242452407 | G:C | 3D | 4068757 | C:G | 1.00 |
| UN | 242452407 | G:C | 3D | 4068759 | G:A | 1.00 |
| UN | 242452407 | G:C | 3D | 4068764 | C:G | 1.00 |
| UN | 242452407 | G:C | UN | 242439365 | G:C | 1.00 |
| UN | 242452407 | G:C | UN | 242439370 | C:T | 1.00 |
| UN | 242452407 | G:C | UN | 242439372 | G:C | 1.00 |
| UN | 242452407 | G:C | UN | 242452400 | G:C | 1.00 |
| UN | 242452407 | G:C | UN | 242452405 | C:T | 1.00 |
| UN | 36153637 | C:T | 3B | 6309968 | G:A | 0.96 |
| UN | 242439372 | G:C | UN | 36153637 | C:T | 0.96 |
| UN | 242452400 | G:C | UN | 36153637 | C:T | 0.96 |
| UN | 242452407 | G:C | UN | 36153637 | C:T | 0.96 |
| UN | 242439365 | G:C | UN | 36153637 | C:T | 0.96 |
| UN | 242439370 | C:T | UN | 36153637 | C:T | 0.96 |
| UN | 242452405 | C:T | UN | 36153637 | C:T | 0.96 |
| UN | 36153637 | C:T | 3B | 6309966 | C:G | 0.96 |
| UN | 36153637 | C:T | 3D | 4068759 | G:A | 0.96 |
| UN | 36153637 | C:T | 3B | 6309973 | C:G | 0.96 |
| UN | 36153637 | C:T | 3D | 4068764 | C:G | 0.95 |
| UN | 36153637 | C:T | 3D | 4068757 | C:G | 0.95 |
| 3B | 6309968 | G:A | 3B | 5601689 | C:A | 0.91 |
| UN | 242439372 | G:C | 3B | 5601689 | C:A | 0.91 |
| UN | 242452400 | G:C | 3B | 5601689 | C:A | 0.91 |
| UN | 242452407 | G:C | 3B | 5601689 | C:A | 0.91 |
| UN | 242439370 | C:T | 3B | 5601689 | C:A | 0.91 |
| UN | 242452405 | C:T | 3B | 5601689 | C:A | 0.91 |
| UN | 242439365 | G:C | 3B | 5601689 | C:A | 0.91 |
| 3B | 6309966 | C:G | 3B | 5601689 | C:A | 0.91 |
| 3D | 4068759 | G:A | 3B | 5601689 | C:A | 0.91 |
| 3B | 6309973 | C:G | 3B | 5601689 | C:A | 0.91 |
| 3D | 909572 | G:T | 3B | 6309968 | G:A | 0.91 |
| UN | 242439372 | G:C | 3D | 909572 | G:T | 0.91 |
| UN | 242452400 | G:C | 3D | 909572 | G:T | 0.91 |
| UN | 242452407 | G:C | 3D | 909572 | G:T | 0.91 |
| UN | 242439370 | C:T | 3D | 909572 | G:T | 0.91 |
| UN | 242452405 | C:T | 3D | 909572 | G:T | 0.91 |
| 3D | 4068764 | C:G | 3B | 5601689 | C:A | 0.91 |
| UN | 242439365 | G:C | 3D | 909572 | G:T | 0.91 |
| 3D | 4068757 | C:G | 3B | 5601689 | C:A | 0.91 |
| 3D | 909572 | G:T | 3B | 6309966 | C:G | 0.91 |
| 3D | 4068759 | G:A | 3D | 909572 | G:T | 0.91 |
| 3D | 909572 | G:T | 3B | 6309973 | C:G | 0.91 |
| 3D | 4068757 | C:G | 3D | 909572 | G:T | 0.91 |
| 3D | 4068764 | C:G | 3D | 909572 | G:T | 0.91 |
| UN | 234960006 | C:T | 3B | 6309968 | G:A | 0.89 |
| UN | 242439372 | G:C | UN | 234960006 | C:T | 0.89 |
| UN | 242452400 | G:C | UN | 234960006 | C:T | 0.89 |
| UN | 242452407 | G:C | UN | 234960006 | C:T | 0.89 |
| UN | 242439370 | C:T | UN | 234960006 | C:T | 0.89 |
| UN | 242452405 | C:T | UN | 234960006 | C:T | 0.89 |
| UN | 242439365 | G:C | UN | 234960006 | C:T | 0.89 |
| UN | 234960006 | C:T | 3B | 6309966 | C:G | 0.89 |
| UN | 234960006 | C:T | 3D | 4068759 | G:A | 0.89 |
| UN | 234960006 | C:T | 3B | 6309973 | C:G | 0.89 |
| UN | 36153637 | C:T | 3B | 5601689 | C:A | 0.89 |
| UN | 234960006 | C:T | 3D | 4068757 | C:G | 0.89 |
| UN | 234960006 | C:T | 3D | 4068764 | C:G | 0.89 |
| UN | 36153637 | C:T | 3D | 909572 | G:T | 0.89 |
| UN | 234960006 | C:T | UN | 36153637 | C:T | 0.87 |
| UN | 34103779 | G:A | 3B | 6309968 | G:A | 0.87 |
| UN | 242439372 | G:C | UN | 34103779 | G:A | 0.87 |
| UN | 242452400 | G:C | UN | 34103779 | G:A | 0.87 |
| UN | 242452407 | G:C | UN | 34103779 | G:A | 0.87 |
| UN | 36153637 | C:T | UN | 34103779 | G:A | 0.87 |
| UN | 242439365 | G:C | UN | 34103779 | G:A | 0.87 |
| UN | 242439370 | C:T | UN | 34103779 | G:A | 0.87 |
| UN | 242452405 | C:T | UN | 34103779 | G:A | 0.87 |
| UN | 34103779 | G:A | 3B | 6309966 | C:G | 0.87 |
| UN | 34103779 | G:A | 3D | 4068759 | G:A | 0.87 |
| UN | 34103779 | G:A | 3B | 6309973 | C:G | 0.87 |
| UN | 34103779 | G:A | 3D | 4068757 | C:G | 0.87 |
| UN | 34103779 | G:A | 3D | 4068764 | C:G | 0.87 |
| UN | 36153637 | C:T | 3B | 10644041 | T:A | 0.77 |
| 3D | 4068764 | C:G | 3B | 10644041 | T:A | 0.76 |
| 3D | 4068757 | C:G | 3B | 10644041 | T:A | 0.76 |
| 3B | 10644041 | T:A | 3B | 6309968 | G:A | 0.75 |
| UN | 242439372 | G:C | 3B | 10644041 | T:A | 0.75 |
| UN | 242452400 | G:C | 3B | 10644041 | T:A | 0.75 |
| UN | 242452407 | G:C | 3B | 10644041 | T:A | 0.75 |
| UN | 242439365 | G:C | 3B | 10644041 | T:A | 0.75 |
| UN | 242439370 | C:T | 3B | 10644041 | T:A | 0.75 |
| UN | 242452405 | C:T | 3B | 10644041 | T:A | 0.75 |
| 3B | 10644041 | T:A | 3B | 6309966 | C:G | 0.74 |
| 3D | 4068759 | G:A | 3B | 10644041 | T:A | 0.74 |
| 3B | 10644041 | T:A | 3B | 6309973 | C:G | 0.74 |
| UN | 234960006 | C:T | 3B | 10644041 | T:A | 0.71 |
| UN | 34103779 | G:A | 3B | 10644041 | T:A | 0.70 |
| 3B | 10644041 | T:A | 3B | 5601689 | C:A | 0.70 |
| 3D | 909572 | G:T | 3B | 10644041 | T:A | 0.70 |
