# Supplementary material for: QTL Analysis of Adult Plant Resistance to Stripe Rust in a Winter Wheat Recombinant Inbred Population
Source: Plants (Basel). 2021 Mar 18;10(3):572. doi: 10.3390/plants10030572 (PMC8002966; doi:10.3390/plants10030572)
Supplement: Supplementary file 1 [file plants-10-00572-s001.zip › Table S4.docx]

Table S4: Stripe rust markers from GrainGenes chromosome 3B with their linkage map location, reference genome location, and associated stripe rust genes. (A) The stripe rust genes and markers are in the order of the GrainGenes linkage map, from 0cM to 17.9cM with a break between 12.2cM and 14.6cM. The locations of the markers as determined by a BLAST search of the IWGSC RefSeq v1.0 reference genome are given in Mb in the last column. (B) Table A rearranged to follow the order of the reference genome location from 0.2Mb to 26.5Mb. Significant SNPs and Subgroups have been added in the last column according to their physical location on the reference sequence.

**A**

| **GrainGenes Stripe Rust Gene Location (cM)** | | | **GrainGenes Location (cM)** | **GrainGenes Marker Name** | **Reference Sequence Location of Marker Match (Mb)** |
| --- | --- | --- | --- | --- | --- |
| *Yr4* | *Yr57* |  | 0.0 |  |  |
|  |  |  | 0.7 | IWB63385 | 26.5 |
|  |  |  | 1.8 |  |  |
|  |  |  | 2.2 | IWB23456 | 5.6 |
|  |  | *Yr30* | 2.3 |  |  |
|  |  |  | 2.8 | IWB4400 | 1.0 |
|  |  |  | 2.8 | IWB5792 | 1.0 |
|  |  |  | 2.8 | IWB32636 | 1.0 |
|  |  |  | 2.9 | IWB60457 | 0.2 |
|  |  |  | 2.9 | IWB56145 | 0.2 |
|  |  |  | 3.0 | IWB62087 | 1.0 |
|  |  |  | 3.0 | IWB48179 | 2.0 |
|  |  |  | 3.7 | IWB7629 | 18.9 |
|  |  |  | 3.7 | IWB12963 | 18.8 |
|  |  |  | 3.8 | IWB4059 | 2.5 |
|  |  |  | 3.8 | IWB12047 | 2.5 |
|  |  |  | 3.8 | IWB4058 | 2.5 |
|  |  |  | 3.8 | IWB23206 | 3.3 |
|  |  |  | 3.8 | IWB34636 | 1.0 |
|  |  |  | 3.8 | IWB11112 | 3.2 |
|  |  |  | 3.8 | IWB11873 | 2.5 |
|  |  |  | 3.8 | IWB13576 | 1.0 |
|  |  |  | 3.8 | IWB24953 | 3.0 |
|  |  |  | 3.8 | IWB32635 | 1.0 |
|  |  |  | 3.8 | IWB41709 | 2.5 |
|  |  |  | 4.2 | IWB30660 | 2.5 |
|  |  |  | 4.8 | IWB6491 | 14.0 |
|  |  |  | 5.3 | *Xgwm533* | 8.5 |
|  |  |  | 5.4 | IWB5790 | 4.1 |
|  |  |  | 5.4 | IWB21932 | 3.3 |
|  |  |  | 5.5 | IWB64176 | 4.1 |
|  |  |  | 5.5 | IWB44132 | 4.4 |
|  |  |  | 5.6 | IWA3271 | 3.4 |
|  |  |  | 5.7 | IWB41429 | 3.9 |
|  |  |  | 5.7 | IWB29578 | 18.7 |
|  |  |  | 5.7 | IWB65213 | 4.3 |
|  |  |  | 5.7 | IWB4522 | 3.4 |
|  |  |  | 5.7 | IWB24960 | 3.4 |
|  |  |  | 5.7 | IWB62540 | 3.6 |
|  |  |  | 5.8 | IWB41804 | 0.2 |
|  |  |  | 6.2 | IWB75019 | 5.6 |
|  |  |  | 6.2 | IWB8884 | 4.5 |
|  |  |  | 6.2 | IWB64989 | 5.6 |
|  |  |  | 6.2 | IWB1428 | 13.8 |
|  |  |  | 6.3 | IWA6038 | 14.0 |
|  |  |  | 6.3 | IWA4796 | 6.0 |
|  |  |  | 6.8 | IWB8756 | 5.7 |
|  |  |  | 6.8 | IWA6471 | 5.7 |
|  |  |  | 6.9 |  |  |
|  |  |  | 7.0 | IWB66050 | 5.9 |
|  |  |  | 7.0 | IWB50465 | 6.7 |
|  |  |  | 7.1 | IWB11923 | 14.0 |
|  |  |  | 7.1 | IWB17659 | 8.7 |
|  |  |  | 7.1 | IWB4668 | 14.0 |
|  |  |  | 7.1 | IWB57993 | 11.1 |
|  |  |  | 7.1 | IWB7464 | 18.2 |
|  |  |  | 7.1 | IWB57529 | 6.7 |
|  |  |  | 7.1 | IWB46712 | 8.3 |
|  |  |  | 7.2 | IWB2086 | 6.2 |
|  |  |  | 7.2 | IWB51224 | 6.7 |
|  |  |  | 7.2 | IWB54306 | 6.7 |
|  |  |  | 7.4 | IWA3103 | 6.7 |
|  |  |  | 7.6 | IWB63732 | 14.0 |
|  |  |  | 7.7 | IWB1836 | 6.3 |
|  |  |  | 7.7 | IWB29466 | 6.2 |
|  |  |  | 7.7 | IWB1837 | 6.3 |
|  |  |  | 8.1 | IWA4654 | 14.0 |
|  |  |  | 8.8 | IWB1170 | 5.8 |
|  |  |  | 8.8 | IWB56857 | 5.6 |
|  |  |  | 8.8 | IWB52306 | 6.0 |
|  |  |  | 8.8 | IWB64711 | 13.8 |
|  |  |  | 9.5 | IWA4801 | 7.2 |
|  |  |  | 9.6 | IWA7230 | 7.2 |
|  |  |  | 10.0 |  |  |
|  |  |  | 10.3 | IWA289 | 16.0 |
|  |  |  | 10.4 | IWB56524 | 14.0 |
|  |  |  | 10.4 | IWB20762 | 5.7 |
|  |  |  | 10.4 | IWB60424 | 13.8 |
|  |  |  | 10.9 | IWB29081 | 7.2 |
|  |  |  | 10.9 | IWB23378 | 6.4 |
|  |  |  | 10.9 | IWB39411 | 12.3 |
|  |  |  | 10.9 | IWB19838 | 12.3 |
|  |  |  | 10.9 | IWB8467 | 6.8 |
|  |  |  | 10.9 | IWA758 | 9.9 |
|  |  |  | 10.9 | IWB49310 | 9.2 |
|  |  |  | 10.9 | IWB60770 | 12.3 |
|  |  |  | 10.9 | IWB61607 | 14.0 |
|  |  |  | 10.9 | IWB6054 | 9.1 |
|  |  |  | 11.2 | IWA7174 | 7.1 |
|  |  |  | 11.9 | IWA5106 | 7.1 |
|  |  |  | 12.2 | IWA4800 | 7.2 |
|  |  |  | 12.2 | IWA2691 | 9.1 |
|  |  |  | 12.2 | IWA5299 | 8.5 |
| ////////////////////// No stripe rust genes in this region //////////////////////// | | | | | |
|  |  |  | 14.6 | IWA2177 | 14.0 |
|  |  |  | 14.7 | IWB13827 | 10.7 |
|  |  |  | 14.7 | IWB32683 | 11.0 |
|  |  |  | 14.8 | IWB45836 | 12.5 |
|  |  |  | 14.8 | IWB60771 | 12.3 |
|  |  | *Yrns-B1* | 14.9 | IWB6299 | 13.8 |
|  |  |  | 15.1 | IWB40172 | 17.1 |
|  |  |  | 15.4 | IWB9212 | 15.5 |
|  |  |  | 15.4 | IWB39782 | 15.0 |
|  |  |  | 15.4 | IWB9415 | 15.5 |
|  |  |  | 15.5 | IWB35202 | 16.4 |
|  |  |  | 15.5 | IWB2094 | 15.0 |
|  |  |  | 15.5 | IWB29378 | 16.4 |
|  |  |  | 15.5 | IWB39254 | 15.0 |
|  |  |  | 15.9 | IWB8773 | 18.2 |
|  |  |  | 16.0 | IWA2493 | 17.1 |
|  |  |  | 16.0 | IWB57184 | 16.5 |
|  |  |  | 16.0 | IWB31375 | 16.5 |
|  |  |  | 16.0 | IWB30033 | 16.4 |
|  |  |  | 16.5 |  |  |
|  |  |  | 17.5 | IWA4340 | 24.9 |
|  |  |  | 17.9 | IWA7231 | 26.6 |
|  |  |  | 17.9 | IWA3260 | 15.0 |
|  |  |  | 17.9 | IWA7678 | 15.0 |

**B**

| **Stripe Rust Gene Location** | | | | **GrainGenes Location (cM)** | **GrainGenes Marker Name** | **Reference Sequence Location of Marker Match (Mb)** | **Significant SNPs and Subgroups of Significant SNPs (Mb)** |
| --- | --- | --- | --- | --- | --- | --- | --- |
|  | *Yr57* | *Yr30* |  | 2.9 | IWB60457 | 0.2 |  |
|  |  |  |  | 2.9 | IWB56145 | 0.2 |  |
|  |  |  |  | 5.8 | IWB41804 | 0.2 |  |
|  |  |  |  | 2.8 | IWB4400 | 1.0 |  |
|  |  |  |  | 2.8 | IWB5792 | 1.0 |  |
|  |  |  |  | 2.8 | IWB32636 | 1.0 |  |
|  |  |  |  | 3.0 | IWB62087 | 1.0 |  |
|  |  |  |  | 3.8 | IWB34636 | 1.0 |  |
|  |  |  |  | 3.8 | IWB13576 | 1.0 |  |
|  |  |  |  | 3.8 | IWB32635 | 1.0 |  |
|  |  |  |  | 3.0 | IWB48179 | 2.0 |  |
|  |  |  |  | 3.8 | IWB4059 | 2.5 |  |
|  |  |  |  | 3.8 | IWB12047 | 2.5 |  |
|  |  |  |  | 3.8 | IWB4058 | 2.5 |  |
|  |  |  |  | 3.8 | IWB11873 | 2.5 |  |
|  |  |  |  | 3.8 | IWB41709 | 2.5 |  |
|  |  |  |  | 4.2 | IWB30660 | 2.5 |  |
|  |  |  |  | 3.8 | IWB24953 | 3.0 |  |
|  |  |  |  | 3.8 | IWB11112 | 3.2 |  |
|  |  |  |  | 3.8 | IWB23206 | 3.3 |  |
|  |  |  |  | 5.4 | IWB21932 | 3.3 |  |
|  |  |  |  | 5.6 | IWA3271 | 3.4 |  |
|  |  |  |  | 5.7 | IWB4522 | 3.4 |  |
|  |  |  |  | 5.7 | IWB24960 | 3.4 |  |
|  |  |  |  | 5.7 | IWB62540 | 3.6 |  |
|  |  |  |  | 5.7 | IWB41429 | 3.9 |  |
|  |  |  |  | 5.4 | IWB5790 | 4.1 |  |
|  |  |  |  | 5.5 | IWB64176 | 4.1 |  |
|  |  |  |  | 5.7 | IWB65213 | 4.3 |  |
|  |  |  |  | 5.5 | IWB44132 | 4.4 |  |
|  |  |  |  | 6.2 | IWB8884 | 4.5 |  |
|  |  |  |  | 2.2 | IWB23456 | 5.6 |  |
|  |  | *Yr30* |  | 6.2 | IWB75019 | 5.6 |  |
|  |  |  |  | 6.2 | IWB64989 | 5.6 |  |
|  |  |  |  | 8.8 | IWB56857 | 5.6 | S3B_5601689 |
|  |  | *Yr30* |  | 6.8 | IWB8756 | 5.7 |  |
|  |  |  |  | 6.8 | IWA6471 | 5.7 |  |
|  |  |  |  | 10.4 | IWB20762 | 5.7 |  |
|  | *Yr57* |  |  | 8.8 | IWB1170 | 5.8 |  |
|  |  |  |  | 7.0 | IWB66050 | 5.9 |  |
|  |  | *Yr30* |  | 6.3 | IWA4796 | 6.0 |  |
|  |  |  |  | 8.8 | IWB52306 | 6.0 |  |
|  |  |  |  | 7.2 | IWB2086 | 6.2 |  |
|  |  |  |  | 7.7 | IWB29466 | 6.2 |  |
|  |  |  |  | 7.7 | IWB1836 | 6.3 |  |
|  |  |  |  | 7.7 | IWB1837 | 6.3 | 3B_Subgroup1, UN_Subgroup1 |
|  |  |  |  | 10.9 | IWB23378 | 6.4 |  |
|  | *Yr57* |  |  | 7.0 | IWB50465 | 6.7 |  |
|  |  |  |  | 7.1 | IWB57529 | 6.7 |  |
|  |  |  |  | 7.2 | IWB51224 | 6.7 |  |
|  |  |  |  | 7.2 | IWB54306 | 6.7 |  |
|  |  |  |  | 7.4 | IWA3103 | 6.7 |  |
|  |  |  |  | 10.9 | IWB8467 | 6.8 |  |
|  |  |  |  | 11.2 | IWA7174 | 7.1 |  |
|  |  |  |  | 11.9 | IWA5106 | 7.1 |  |
|  | *Yr57* |  |  | 9.5 | IWA4801 | 7.2 |  |
|  |  |  |  | 9.6 | IWA7230 | 7.2 |  |
|  |  |  |  | 10.9 | IWB29081 | 7.2 |  |
|  |  |  |  | 12.2 | IWA4800 | 7.2 |  |
|  | *Yr57* |  |  | 7.1 | IWB46712 | 8.3 |  |
|  |  | *Yr30* |  | 5.3 | *Xgwm533* | 8.5 |  |
|  |  |  |  | 12.2 | IWA5299 | 8.5 |  |
|  | *Yr57* |  |  | 7.1 | IWB17659 | 8.7 |  |
|  |  |  |  | 10.9 | IWB6054 | 9.1 |  |
|  |  |  |  | 12.2 | IWA2691 | 9.1 |  |
|  |  |  |  | 10.9 | IWB49310 | 9.2 |  |
|  |  |  |  | 10.9 | IWA758 | 9.9 | S3B_10644041 |
|  |  |  |  | 14.7 | IWB13827 | 10.7 |  |
|  |  |  |  | 14.7 | IWB32683 | 11.0 |  |
|  | *Yr57* |  |  | 7.1 | IWB57993 | 11.1 | SUN_36153637 |
|  |  |  |  | 10.9 | IWB39411 | 12.3 |  |
|  |  |  |  | 10.9 | IWB19838 | 12.3 |  |
|  |  |  |  | 10.9 | IWB60770 | 12.3 |  |
|  |  |  |  | 14.8 | IWB60771 | 12.3 |  |
|  |  |  |  | 14.8 | IWB45836 | 12.5 |  |
|  | *Yr57* | *Yr30* |  | 6.2 | IWB1428 | 13.8 |  |
|  |  |  |  | 8.8 | IWB64711 | 13.8 |  |
|  |  |  |  | 10.4 | IWB60424 | 13.8 |  |
|  |  |  | *Yrns-B1* | 14.9 | IWB6299 | 13.8 |  |
|  | *Yr57* | *Yr30* |  | 4.8 | IWB6491 | 14.0 |  |
|  |  |  |  | 6.3 | IWA6038 | 14.0 |  |
|  |  |  |  | 7.1 | IWB11923 | 14.0 |  |
|  |  |  |  | 7.1 | IWB4668 | 14.0 |  |
|  |  |  |  | 7.6 | IWB63732 | 14.0 |  |
|  |  |  |  | 8.1 | IWA4654 | 14.0 |  |
|  |  |  |  | 10.4 | IWB56524 | 14.0 |  |
|  |  |  |  | 10.9 | IWB61607 | 14.0 |  |
|  |  |  |  | 14.6 | IWA2177 | 14.0 |  |
|  |  |  | *Yrns-B1* | 15.4 | IWB39782 | 15.0 |  |
|  |  |  |  | 15.5 | IWB2094 | 15.0 |  |
|  |  |  |  | 15.5 | IWB39254 | 15.0 |  |
|  |  |  |  | 17.9 | IWA3260 | 15.0 |  |
|  |  |  |  | 17.9 | IWA7678 | 15.0 |  |
|  |  |  | *Yrns-B1* | 15.4 | IWB9212 | 15.5 |  |
|  |  |  |  | 15.4 | IWB9415 | 15.5 |  |
|  |  |  |  | 10.3 | IWA289 | 16.0 |  |
|  |  |  | *Yrns-B1* | 15.5 | IWB35202 | 16.4 |  |
|  |  |  |  | 15.5 | IWB29378 | 16.4 |  |
|  |  |  |  | 16.0 | IWB30033 | 16.4 |  |
|  |  |  |  | 16.0 | IWB57184 | 16.5 |  |
|  |  |  |  | 16.0 | IWB31375 | 16.5 |  |
|  |  |  |  | 15.1 | IWB40172 | 17.1 |  |
|  |  |  |  | 16.0 | IWA2493 | 17.1 |  |
|  | *Yr57* |  |  | 7.1 | IWB7464 | 18.2 |  |
|  |  |  | *Yrns-B1* | 15.9 | IWB8773 | 18.2 |  |
|  | *Yr57* | *Yr30* |  | 5.7 | IWB29578 | 18.7 |  |
|  |  |  |  | 3.7 | IWB12963 | 18.8 |  |
|  |  |  |  | 3.7 | IWB7629 | 18.9 |  |
|  |  |  |  | 17.5 | IWA4340 | 24.9 |  |
| *Yr4* | *Yr57* |  |  | 0.7 | IWB63385 | 26.5 |  |
|  |  |  |  | 17.9 | IWA7231 | 26.6 |  |
