# Supplementary material for: QTL Analysis of Adult Plant Resistance to Stripe Rust in a Winter Wheat Recombinant Inbred Population
Source: Plants (Basel). 2021 Mar 18;10(3):572. doi: 10.3390/plants10030572 (PMC8002966; doi:10.3390/plants10030572)
Supplement: Supplementary file 1 [file plants-10-00572-s001.zip › Table S6.docx]

**Table S6:** Unvalidated KASP marker designs for each of the four markers needed to retain all resistance alleles on chromosome 3B and of the two markers needed to retain all resistance alleles on chromosome 3D. ‘Location of Sequence’ is the physical location of the genomic sequence (in Ensembl Plants notation) shown in the last column. The ‘Significant SNPs Covered’ are the SNPs that have an LD of 1.0 with the marker SNP. ‘Sequence’ shows the syntax that should be used when ordering KASP markers for polyploid organisms. <N> denotes the anchor point for the KASP design process, where the nucleotide differs in the desired genome from the two other genomes of hexaploid wheat. [N/N] denotes the SNP location of interest with the susceptible and resistant alleles noted. These markers have not yet been validated and may need further adjustments.

| **SNP Used for Marker** | **Location of Sequence** | **Significant SNPs Covered** | **Sequence** |
| --- | --- | --- | --- |
| 3B_5601689 | 3B:5601629-5601717 | S3B_5601689 | TTACCATGAAACACTTA<G>AACGAAAATAAAATCTTGCTTCTTGTACTCTGCACTGCAGAA[A/C]ATGCAGAAATTCTTGGAATTTGCCGCGT |
| 3B_6309966 | 3B:6309924-6309999 | S3B_6309966  S3B_6309968  S3B_6309973  SUN_242439365  SUN_242439370  SUN_242439372  SUN_242452400  SUN_242452405  SUN_242452407 | CCTGAATCTCAAGCCTCATTTGGGGCAACGAATCGAAGCCCA[C/G]CAAGCA<G>CATAACAAGAACGAAAATGGTGAGTG |
| 3B_10644041 | 3B:10643983-10644064 | S3B_10644041 | TTATAAATACTGTTGCATGCATGG<C>AGTAGATGCTCACTGGCGAATGGTAACGACCCA[A/T]GTTGCGAGCGAACTACAGTACTG |
| UN_36153637 | 3B:554945869-554945957 | SUN_36153637 | ATCCACAAGCAAGGAGGAGAAGGCCGAC<A>TCTAAGGCTGCACCATCCTC[T/C]ATCCGCGATGAGGGAAGAGCAAATTCATGACGTCCATCT |
| 3D_909572 | 3D:909522-909607 | S3D_909572  SUN_34103779  SUN_234960006 | GCCTAACGTACCTTCAAGAACCACGCGGCAA<G>TTCCAAGAATTTCTGCAT[T/G]TTCTGCAGTGCAAGTACAGGAAACAAGATTTTATT |
| 3D_4068757 | 3D:4068718-4068804 | S3D_4068757  S3D_4068759  S3D_4068764 | GAATCTCAAGCCTCATTTGGGGCAACGAATCCAAGCCAT[C/G]TAAGCACCATAATAAGAA<T>GAAAATGGCGATTCGCAGAGTGCATACA |
